# Mapping the developmental structure of stereotyped and individual-unique behavioral spaces in *C. elegans*

**DOI:** 10.1016/j.celrep.2024.114683

**Published:** 2024-08-27

**Authors:** Yuval Harel, Reemy Ali Nasser, Shay Stern

**Affiliations:** 1 Faculty of Biology, Technion - Israel Institute of Technology, Haifa, Israel

**Keywords:** behavior, developmental biology, individuality, neuromodulation, *C. elegans*

## Abstract

Developmental patterns of behavior are variably organized in time and among different individuals. However, long-term behavioral diversity was previously studied using pre-defined behavioral parameters, representing a limited fraction of the full individuality structure. Here, we continuously extract ∼1.2 billion body postures of ∼2,200 single *C. elegans* individuals throughout their full development time to create a complete developmental atlas of stereotyped and individual-unique behavioral spaces. Unsupervised inference of low-dimensional movement modes of each single individual identifies a dynamic developmental trajectory of stereotyped behavioral spaces and exposes unique behavioral trajectories of individuals that deviate from the stereotyped patterns. Moreover, classification of behavioral spaces within tens of neuromodulatory and environmentally perturbed populations shows plasticity in the temporal structures of stereotyped behavior and individuality. These results present a comprehensive atlas of continuous behavioral dynamics across development time and a general framework for unsupervised dissection of shared and unique developmental signatures of behavior.

## Highlights

• Construction of a complete developmental atlas of *C. elegans* behavior
• Unsupervised inference of stereotyped and individual-unique behavioral spaces
• Identified gradual dynamics of stereotyped behavior and long-term individual differences
• Neuronal and environmental perturbations reveal plasticity of behavioral spaces

## Graphical abstract

**Figure:**
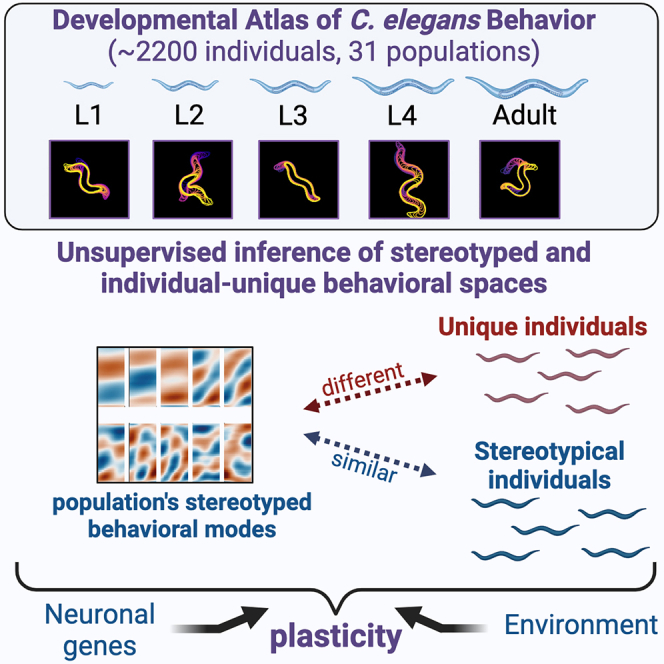

## Teaser

Harel et al. construct a complete atlas of *C. elegans* behavioral spaces by continuously monitoring the posture dynamics of ∼2,200 individuals across their entire developmental trajectory. They find gradual changes in stereotyped behavior and individual uniqueness over time. Comparing various populations, they show time-dependent plasticity in the developmental trajectories of behavioral spaces.

## Introduction

Complex patterns of behavior may be fundamentally described as the composition of underlying movement states that integrate with different intensities and variable temporal order to form high-level manifestations of behavior. In particular, these underlying modes of movement, mainly characterized by posture changes during specific developmental windows, were shown across species to form stereotyped movement patterns that are shared by many individuals within the population, such as in *Caenorhabditis elegans*,^1^^,^^2^^,^^3^^,^^4^ *Drosophila melanogaster*,^5^^,^^6^ and mice.^7^^,^^8^^,^^9^ However, the complete and continuous organization of stereotyped modes of posture dynamics across and within all developmental stages of an organism is still underexplored. In addition, it has been shown that animals within the same population, even when isogenic and grown in the same environment, show long-term individual-to-individual behavioral diversity that distinguish them from one another,^10^^,^^11^^,^^12^^,^^13^^,^^14^^,^^15^ including across developmental timescales.^16^^,^^17^ These inter-individual differences were extracted mainly by using pre-defined behavioral parameters, exposing a limited set of behavioral characteristics for identifying individuality within populations.

Here, we used unsupervised inference of the complete dynamic structure of *C. elegans* behavior from egg hatching to adulthood to define a developmental trajectory of behavioral spaces of each single individual within the population. The nematode *C. elegans* is an ideal system to study the temporal and inter-individual variation in posture dynamics among isogenic individuals and across developmental timescales due to their short development time of 2.5 days and the homogeneous populations generated by the self-fertilizing reproduction mode of the hermaphrodite. By utilizing a long-term imaging system at high spatiotemporal resolution for continuous behavioral monitoring and posture extraction of multiple isolated individuals, we generated a comprehensive atlas of *C. elegans* behavioral spaces throughout a full developmental trajectory. The dataset includes a total of over 1.2 billion body postures continuously extracted from ∼2,200 individuals across and within all stages of development. Unsupervised inference of the low-dimensional representation of each individual’s behavioral spaces of posture dynamics modes across developmental windows identified both stereotyped and individual-unique behavioral trajectories that dynamically change as development progresses. Moreover, analysis of stereotyped and individual-specific behavioral spaces across tens of neuromodulatory- and environmentally perturbed populations showed widespread plasticity in the temporal organization of shared and unique developmental patterns of behavior.

Overall, the complete atlas of behavioral dynamics throughout development and the unsupervised identification of long-term trajectories of behavioral spaces provide a general framework for inferring diversity in behavioral structures across developmental timescales, at the population and individual level.

## Results

### A complete developmental atlas of *C. elegans* behavioral spaces of posture dynamics

**Figure 1:**
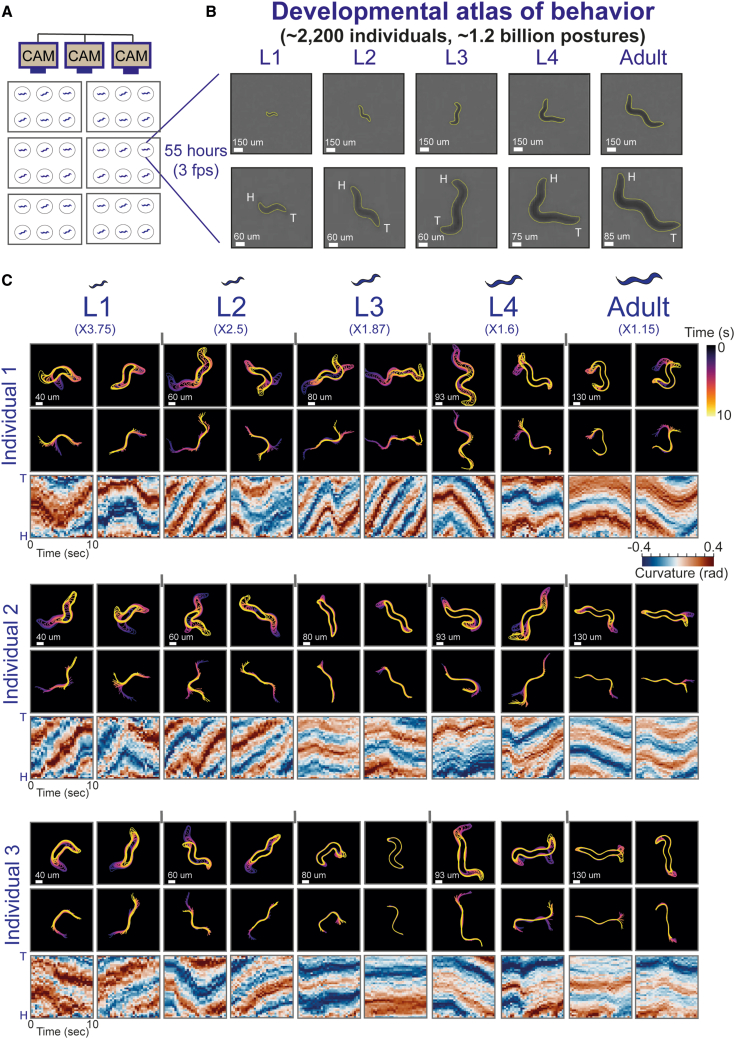
Construction of a complete atlas of *C. elegans* behavioral spaces across development by long-term monitoring of posture dynamics (A) A custom-made multi-camera imaging system allows longitudinal monitoring of posture dynamics of multiple individual worms during their behavior, across and within all stages of development, at high spatiotemporal resolution and under tightly controlled environments. (B) Representative camera images (top) and enlarged images (bottom) of a single individual across all stages of development (L1–L4 and adulthood) and their corresponding computed contours (yellow). Images were enlarged 2.5× for the L1–L3 images, 2× for the L4 image, and 1.75× for the adult image for visual clarity. “H” and “T” indicate head and tail, respectively. Scale bars are indicated in white for each developmental stage. (C) Examples of 10-s posture dynamics windows of three different individuals, during all stages of development. For each individual represented, body contour sequences (top), midline sequences (center), and curvature profiles (head to tail) across 37 points, homogenously distributed along the individual’s midline (bottom). Color code of body contours and midlines marks time progression within the time window. The color code of midline curvature profiles marks the curvature in each point of the midline (head to tail). Posture dynamics images were enlarged for visual clarity (L1: 3.75×, L2: 2.5×, L3: 1.87×, L4: 1.6×, adult: 1.15×). Scale bars are indicated in white for each developmental stage.

We developed an analysis system for studying the complete stereotyped and individual-unique behavioral spaces of *C. elegans* by longitudinal monitoring of the posture dynamics of single individuals during their full development time. The analysis framework is based on a long-term multi-camera imaging system that was previously utilized to extract pre-defined locomotory parameters by solely tracking the individual’s position in the arena as a point in space.^16^^,^^17^ In particular, in this imaging system, single individuals are continuously tracked in isolation from egg hatching to 16 h of adulthood (∼2.5 days), in custom-made laser-cut multi-well plates, and behavioral monitoring is performed at high spatiotemporal resolution and in a tightly controlled environment (Figure 1A).

To represent the complete behavioral space of the spontaneous locomotory movements throughout a full developmental trajectory, we tracked each individual’s body position at sub-second resolution across the days of development. In each frame (total of ∼600,000–700,000 frames per individual), we automatically analyzed a small sub-region around the tracked position, allowing us to capture the individual’s body image (Figure 1B). We then automatically identified the body posture of each individual at each frame by processing the image to detect the contour and midline of the worm (Figures 1B, 1C, and S1A). During *C. elegans* development, the size of individual animals increases significantly from the first larval stage (L1) to adulthood (∼5-fold in length, Figure S1B). To further represent the individual’s pose over time, in each frame we divided the body midline into 40 segments of equal length and quantified the curvature between each pair of adjacent segments (Figures 1C and S1A) (see STAR Methods).^1^ The representation of body postures by using a similar number of midline segments across development allows comparing posture dynamics at different developmental windows and across different individuals within populations (Figure 1C). The long timescale of the behavioral experiments imposes data analysis challenges such as the ability to efficiently identify head-tail direction of individuals across an extremely large set of analyzed frames during development, as well as to maintain the correct alignment across frames in which body posture identification has failed. We developed a computationally efficient method for fast head and tail detection across all developmental stages in the large dataset of individuals (based on differences in side-to-side movements) and for aligning the individual’s midline orientation over development time (Figures 1C, S1C, and S1D) (see STAR Methods).^2^

In total, we analyzed 2,199 individuals (across different genotypes and conditions) continuously during all developmental windows, resulting in a dataset of ∼1.2 billion sequential body postures that are integrated in time to generate the full repertoire of individual movements throughout development. In summary, we have constructed a behavioral atlas and efficient analyses methods for studying the complete developmental dynamics of behavior across *C. elegans* individuals.

### Unsupervised inference of the organization of stereotyped behavioral spaces across development

The continuous long-term quantification of posture dynamics of a large set of individuals across all developmental windows (Figure 1) allows the classification of the underlying dominant modes of movement states throughout the progression of development at the population and individual level. It has been previously demonstrated in various organisms,^5^^,^^7^ including in *C. elegans*,^1^^,^^3^^,^^4^ that temporal sequences of body postures may be utilized for characterizing underlying behavioral states. However, whether individuals show distinct spectrums of stereotyped behavioral modes of posture dynamics at different time windows as they continuously develop, and how these stereotyped behavioral patterns that are shared by individuals organized within and across all developmental stages is still underexplored.

**Figure 2:**
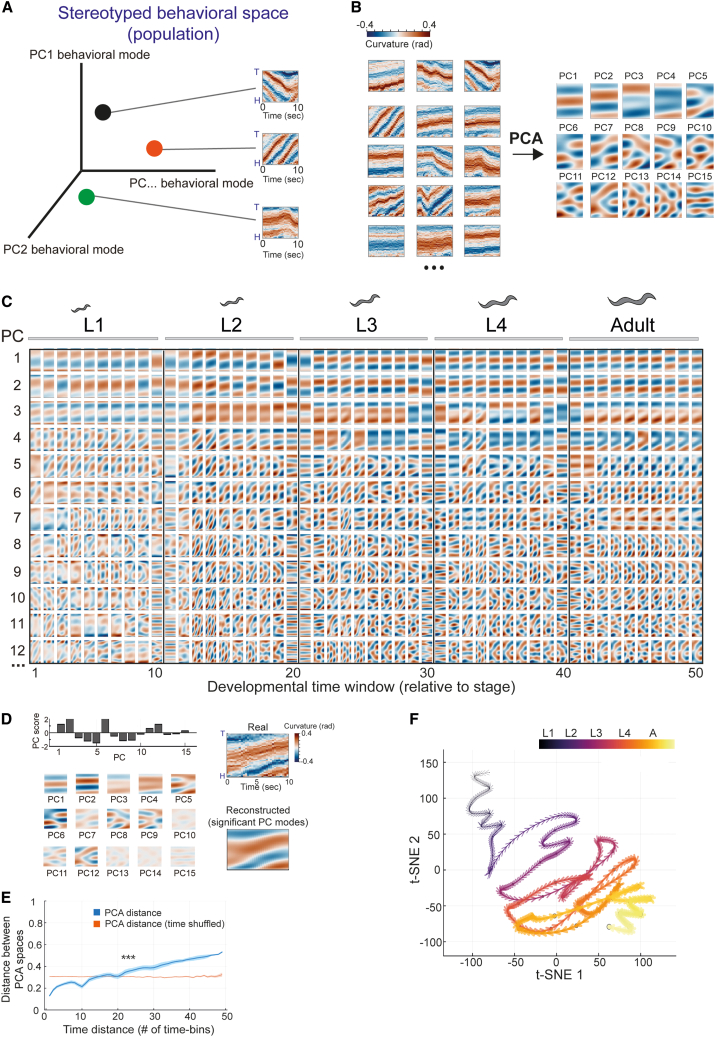
Unsupervised classification of stereotyped behavioral spaces across development (A) A schematic of a stereotyped behavioral space of the population in which posture dynamics windows of individuals are represented as points within the space and are defined by a combination of the behavioral space dimensions, representing the population’s dominant behavioral modes. (B) Representative pool of posture dynamics time windows of wild-type individuals (left) and PC1–PC15 generated by PCA of all posture dynamics windows within the wild-type population (*n* = 123) in a specific developmental window (L4 stage, 7th time bin, explaining 73% of the variation) (right). Posture dynamics windows in (A) and (B) are represented as curvature profiles (head to tail) across 37 points, homogenously distributed along the individual’s midline. (C) Stereotyped developmental trajectory of PCA spaces across developmental time windows. Shown are PC1–PC12 generated by the PCA. The variations of PCs across developmental time windows are indicated in Figures S2D and S2E. (D) An example of 10-s posture dynamics (top right) of an individual and its reconstruction (bottom right) using a combination of PC vectors (bottom left, shown here multiplied by their scores), based on their PC scores (top left). (E) Average distance across stereotyped PCA spaces of the wild-type population separated by a specific number of time windows (blue) relative to average distance across stereotyped PCA spaces that are shuffled in time (1,000 runs, orange). Shaded area represents SEM. The *p* value was generated by bootstrapping (see STAR Methods) for the difference in correlation (Pearson) between the real and time-shuffled dataset. ^∗∗∗^*p* < 0.001. (F) t-SNE representation of distances between stereotyped PCA spaces across development (10 per stage) (see STAR Methods). Each dot represents a stereotyped behavioral space of the wild-type population at a specific developmental window. Color code marks time progression across development (t-SNE perplexity 20).

To study how longitudinal patterns of stereotyped behavioral modes are temporally organized during the developmental trajectory, we sought to first define a stereotyped behavioral space of the wild-type population (*n* = 123) at each developmental window across all stages (Figure 2A). In particular, each behavioral space is defined by its axes (dimensions), which represent the underlying movement modes that are dominant within the population. All movement patterns of individuals could be then represented as points within this space, where each point (a real movement pattern) is defined by a specific combination of the underlying dominant behavioral modes (Figure 2A). To generate a behavioral space in each developmental window, we first age-normalized individuals by dividing the full developmental trajectory of each individual to 50 developmental time windows (10 per stage) based on its lethargus episodes of inactivity during molting, which robustly mark transitions between developmental stages^16^^,^^18^ (Figure S2A). We then quantified all overlapping 10-s sequences of body postures of all individuals in each of the 50 developmental time windows (400,000–2 million posture sequences per developmental time window). This analysis results in 50 pools of high-resolution body movements within the wild-type population, which correspond to the 50 developmental windows across and within all stages. Our approach of analyzing the complete temporal sequence of body postures within time windows allows us to efficiently represent the large spectrum of complex and fast behavioral dynamics shown by *C. elegans* during development, which may be otherwise hard to uniquely capture by averaging curvature or speed in midline points within these different time windows (Figure S2B). To take an unsupervised approach to identifying the underlying stereotypic modes of posture dynamics in each of the 50 developmental time windows, we performed principal-component analysis (PCA) of the pool of all overlapping 10-s body movements of the wild-type individuals (Figures 2B and 2C). To further identify the effective dimensionality (number of significant PC dimensions) in each of the developmental time windows, we used cross-validation of PCA reconstruction errors (see STAR Methods) (Figures S2C and S2D). In particular, the PCA dimensionality reduction method detected PC dimensions that represent underlying dominant modes of posture dynamics that can reconstruct (based on PC scores) each “real” 10-s movement of each individual during a specific developmental time window (Figure 2D). We found that the number of significant PC dimensions detected by the cross-validation method increased over development time, with a relatively lower number of significant PC dimensions detected during earlier developmental stages compared to later stages (average number of significant dimensions: 5.7, 9.6, 12.3, 14.1, 15.6 across the 5 L1-adult developmental stages, respectively) (Figure S2D). Furthermore, the composition of significant PC dimensions across developmental windows showed a spectrum of stereotyped behavioral spaces that are temporally distinct, implying that the population moves through a sequence of non-fixed behavioral spaces during development (Figures 2C and 2F). Interestingly, by quantifying distances between stereotyped behavioral spaces that are exposed by the population at different developmental windows, we found that the distances between PCA behavioral spaces that are closer in time tend to be smaller and vice versa (Figure 2E) (see STAR Methods). Similar analyses of time-shuffled behavioral spaces did not show this dependence (Figure 2E). Furthermore, construction of PCA behavioral spaces using posture dynamics windows of variable length (5–40 s) showed similar results (Figure S2F). These analyses imply a gradual smooth progression of stereotyped behavior throughout development time. A t-distributed stochastic neighbor embedding (t-SNE) map that was constructed for visualization recaptured the relatively higher similarity (smaller distance) between behavioral spaces that are adjacent in time across the developmental trajectory (Figures 2F and S2G).

As a complementary approach, we characterized the stereotyped patterns of posture dynamics by constructing a single PCA space based on the pool of all overlapping posture dynamics windows across the full developmental trajectory (see STAR Methods) (Figure S2H). We then quantified the fraction of variation explained by the identified PCs in each of the 50 time bins to characterize the profile of explored PC modes in each developmental window (see STAR Methods). We found that similar to the space-to-space distances quantified across developmental time windows (Figures 2E and S2F), distances between profiles of PC variations within the same PCA space tend to be smaller between developmental windows that are closer in time (Figure S2I).

In summary, these results show distinct structures of underlying behavioral modes in *C. elegans* across and within developmental windows, defining a dynamic developmental trajectory of behavioral spaces.

### Individual-specific spaces of underlying posture dynamics modes identify behavioral uniqueness

**Figure 3:**
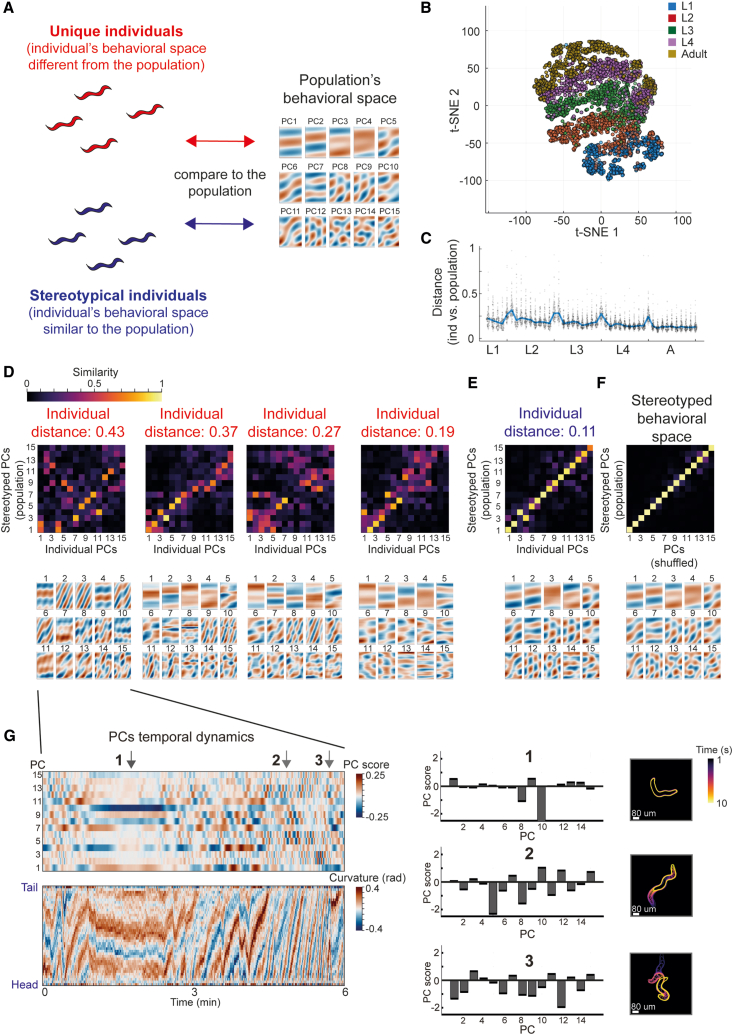
Identifying individual-unique behavioral spaces within populations (A) A schematic representing the comparison of the behavioral space of each single individual to the stereotyped behavioral space of the population during a specific developmental window. These comparisons allow the identification of both unique and stereotypical individuals (different or similar to the behavioral space of the population, respectively). (B) t-SNE representation of distances between individual-specific behavioral spaces of wild-type individuals across developmental time windows. Each dot represents an individual-specific behavioral space at a specific developmental window. (C) Distributions of distances between PCA behavioral spaces of wild-type individuals and the stereotyped PCA behavioral space of the population from mid-L1 stage to adulthood. Each dot represents a single individual. The blue line marks the median distance across the population. (D and E) Examples of similarity matrices between the individual’s and the population’s stereotyped PC modes (top), and the corresponding individual’s PC modes (bottom), in animals that showed high behavioral uniqueness (D) or stereotyped behavior (E) during a specific developmental time window (5th time bin in the L3 stage). (F) Example of a similarity matrix between a PCA behavioral space generated from a shuffled dataset (see STAR Methods) and the population’s stereotyped PC modes (top) and the corresponding stereotyped PC modes of the population (bottom) during a specific developmental time window (5th time bin in the L3 stage). Color code in (D)–(F) marks similarity between PC modes, quantified as the absolute value of the dot product (0–1). (G) Left: example of temporal dynamics of scores of PC modes (top) and the corresponding posture dynamics (bottom) during a 6-min window of the highly unique individual presented in (D). Right: bar plots of scores of PC modes and the corresponding locomotory pattern during three 10-s time windows (indicated by numbers) within the larger window of temporal dynamics represented at left. Scale bars are indicated in white.

Individuals within populations, even when genetically identical and exposed to the same environment, show wide inter-individual behavioral variation,^10^^,^^11^^,^^13^^,^^14^^,^^15^ including across developmental timescales.^16^^,^^17^ An open question is whether unsupervised inference of behavioral spaces of posture dynamics can be used to systematically capture individual-to-individual behavioral diversity at specific developmental windows. We hypothesized that individuals within the population may explore behavioral spaces that are composed of underlying behavioral modes that are different from the dominant modes expressed within the population. Thus, a direct comparison between the individual-specific behavioral space and the population’s stereotyped space would permit the identification of individuals that are behaviorally unique (different from the population), as well as stereotypical individuals that show high behavioral similarity to the population (Figure 3A). We extracted the levels of behavioral uniqueness of individuals within the wild-type population by performing PCA of posture dynamics modes separately for each animal at each developmental window, such that each individual is represented by its own sequence of 50 low-dimensional behavioral spaces throughout development time (Figures 3B and S3A). We then directly compared the behavioral space of each individual at each developmental window to the population’s stereotyped behavioral space (Figures 3C–3F and S3B–S3D). In particular, we first quantified the distance of each of the individual’s PC modes of posture dynamics (represented by the loading vectors of the PCs) to the space spanned by the population’s stereotyped PC modes. Then, based on the integration of these PC distances, we defined a global relative-distance parameter (0–1, low to high uniqueness), which robustly captures the uniqueness of the complete behavioral space of each single animal (see STAR Methods). By systematically quantifying the relative distances between the behavioral spaces of all wild-type individuals and the stereotyped space at each developmental window (Figure 3C), we identified specific individuals that showed variable levels of behavioral difference. For instance, we identified multiple individuals within the population that showed extremely high behavioral uniqueness (large distance parameter) at specific developmental windows (unique individuals) (Figures 3D, 3F, and S3B), implying that their underlying dominant behavioral modes of posture dynamics significantly differ from the behavioral modes of the population. In contrast, other individuals within the same population showed extremely low behavioral uniqueness parameters (closer to 0), implying that most of their individual-specific PC modes of posture dynamics are highly similar to the stereotyped behavioral modes of the population (stereotypical individuals) (Figures 3E, 3F, and S3D). Furthermore, we also detected individuals with intermediate levels of behavioral uniqueness such as animals that showed a high similarity of only a limited fraction of their underlying PC modes to the stereotyped PC modes of the population (Figure S3C), suggesting an overall continuum of behavioral spaces uniqueness that is exposed by the unsupervised method.

Once extreme individuals are detected within the population by the analysis of uniqueness of PCA spaces (Figure 3D), the simultaneous representation of scores of underlying PC modes and of “real” locomotory patterns over time allows the further extraction of unique locomotory movements of these extreme individuals and the study of how these unique patterns are built from specific underlying PC modes (Figure 3G). Interestingly, the temporal analysis showed that while relatively inactive states of locomotory patterns that are enriched within unique individuals may be reconstructed sparsely by a low number of dominant individual-specific PC modes, more complex patterns of these unique individuals are built from many dominant PC modes that are concurrently represented and integrated into the higher-level locomotory movement (Figure 3G). While the indicated examples represent only a small subset of the unique locomotory patterns within the population, overall, these results demonstrate the use of unsupervised inference of individual-specific behavioral spaces for the unbiased detection of individual diversity within populations.

### Long-term individuality in uniqueness of behavioral spaces across developmental windows

**Figure 4:**
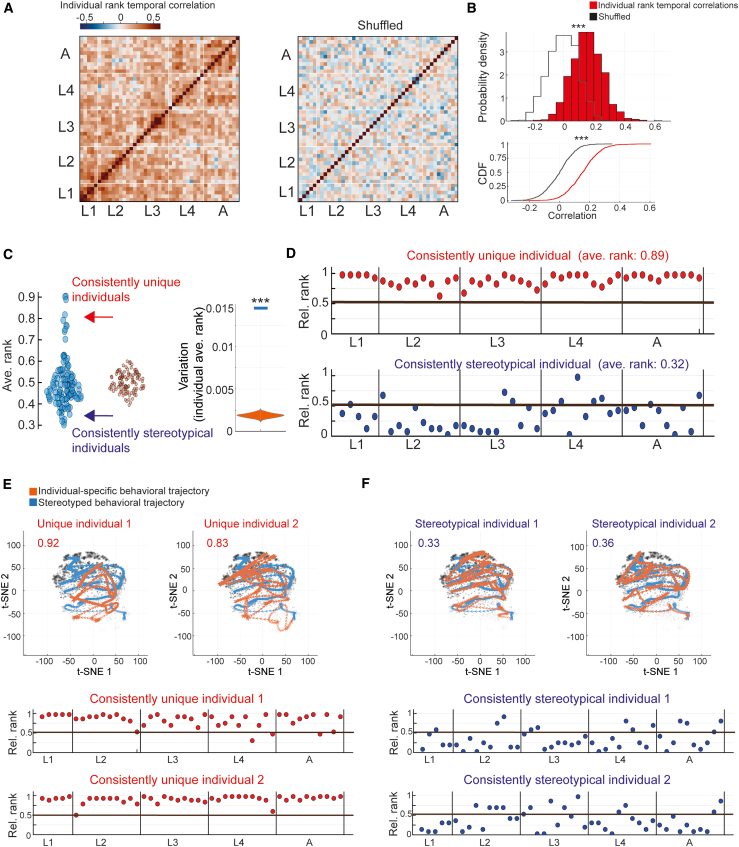
Consistent individuality in behavioral spaces uniqueness across and within developmental stages (A) Left: temporal correlations (Pearson) between relative uniqueness rank of behavioral spaces from the mid-L1 stage to adulthood (45 developmental windows) of wild-type individuals with a full trajectory of PCA spaces (see STAR Methods). Right: temporal correlations between relative uniqueness rank of behavioral spaces generated from a shuffled dataset. Color code marks individual rank temporal correlations. (B) Distributions (top) and their corresponding cumulative distribution function plots (bottom) of total temporal correlations (Pearson) between relative uniqueness rank of individuals (red) and of temporal correlations between individuals generated from a shuffled rank dataset (black) as in (A). ^∗∗∗^*p* < 0.001 (comparison to 1,000 shuffled datasets) (see STAR Methods). (C) Left: distribution of average relative rank of behavioral space uniqueness within the wild-type population (*n* = 112) across developmental windows of individuals (blue) compared to a distribution of average relative rank generated from a shuffled dataset (orange). Each dot represents a single individual within the population. Right: variation in average relative rank of behavioral spaces uniqueness among wild-type individuals across developmental windows (blue bar) compared to variation generated from a shuffled rank dataset (1,000 runs, orange). ^∗∗∗^*p* < 0.001. (D) Relative uniqueness rank of individual-specific behavioral spaces across developmental windows in a consistently unique individual (top, red) and a stereotypical individual that is similar to the population (bottom, blue). The black line marks the median relative rank across the population. (E and F) Top: t-SNE representations of individual-specific time trajectories of behavioral spaces across development (orange) compared to the stereotyped trajectory of the wild-type population (blue) in consistently unique (E) and stereotypical (F) individuals. The gray dots in the background represent all individual-unique behavioral spaces as in Figure 3B. Average relative uniqueness rank of each individual across development is indicated in the top left corner. Bottom: relative uniqueness rank of the individual-specific behavioral spaces across developmental windows of the presented individuals.

To study how long-term individuality signatures of posture dynamics are organized across developmental timescales, we analyzed the consistency in uniqueness of individual-specific behavioral spaces across a full developmental trajectory, within and across all developmental stages. Specifically, we asked whether individuals that show high or low uniqueness of their behavioral space of posture dynamics (Figure 3) will also tend to show similar levels of uniqueness during other developmental windows. To analyze long-term consistency in relative behavioral uniqueness levels, we first ranked all wild-type individuals based on the distance between their individual-specific behavioral spaces and the population’s stereotyped space, in each developmental window, compared to all other individuals within the same experiment (relative rank: 0–1, from most stereotypical to most unique individual in the population; see STAR Methods) (Figure S4A). We then quantified the temporal correlations between individual uniqueness ranks across developmental time windows, such that higher temporal correlations would indicate a higher consistency of individuals in being more or less unique across different developmental periods (Figure 4A). We found that the total temporal correlations between individuals’ uniqueness ranks across the full developmental trajectory were significantly higher compared to a shuffled rank dataset (Figures 4A, 4B, and S4A), implying long-term consistency in levels of behavioral spaces uniqueness throughout development time. Moreover, by separately analyzing temporal correlations within each developmental stage and across all pairs of developmental stages, we found that while individuals show variable consistency levels across different developmental periods, temporal correlations were still highly significant within and across all developmental stages (Figure S4B).

To further utilize the unsupervised detection of behavioral spaces uniqueness for identifying specific individuals within the population that showed an extreme tendency toward being unique or stereotypical throughout development, we quantified each individual’s average uniqueness rank across all developmental windows (Figure 4C). In addition, we analyzed inter-individual variation in average uniqueness rank within the population as a global measure of how extreme individuals are toward being behaviorally unique or stereotypical over development time (Figure 4C). We found that individuals within the wild-type population tended to be more extremely unique or stereotypical across developmental windows, compared to a shuffled rank dataset (Figure 4C). Analysis of specific individuals that had extreme average rank levels showed long-term consistency in exposing high or low uniqueness of behavioral spaces during most of the developmental windows (Figures 4D–4F, S4D, and S4E). Interestingly, individuals that showed extreme uniqueness across development had significantly larger behavioral variation among them as compared to the population (Figure S4C), implying that they do not form a behaviorally homogeneous group but rather that their underlying behavioral modes are substantially variable. Similar time consistency in uniqueness levels was obtained when constructing the PCA behavioral spaces using posture dynamics windows of variable lengths (5–40 s) (Figure S4F) or representing individuals within the same shared PCA behavioral space (see STAR Methods) (Figure S4G). In addition, while lethargus periods of inactivity may be variable across individuals (Figure S5A), time consistency in uniqueness levels was similar when developmental windows that include these lethargus periods (time windows at the start and end of each stage) were excluded from the analysis (Figure S5A). These results suggest the developmental organization of unique behavioral spaces of posture dynamics into consistent individuality signatures.

### Unsupervised detection of plasticity in stereotyped trajectories of behavioral spaces

**Figure 5:**
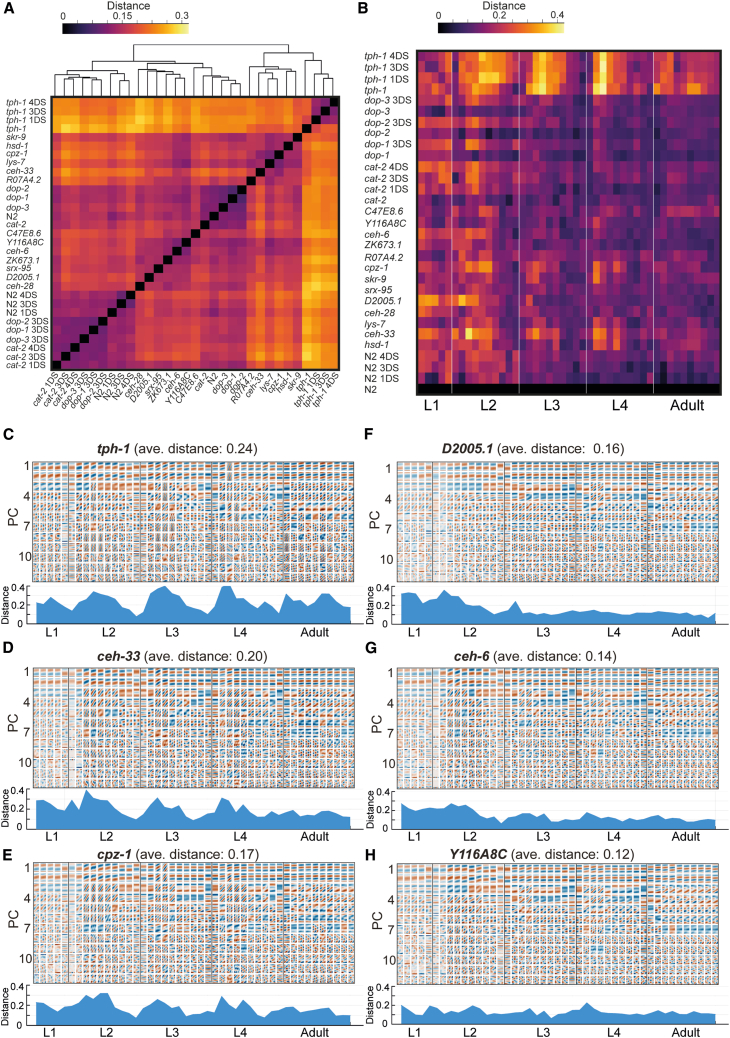
Divergence in stereotyped trajectories of behavioral spaces across mutant and environmentally perturbed populations (A) Hierarchical clustering analysis of 31 analyzed populations (total of 2,199 individuals), including mutants for neuronal genes and environmentally perturbed populations. “DS” indicates days of starvation. Color code indicates relative distance between populations (see STAR Methods) averaged across developmental time windows. (B) Heatmap shows distances between stereotyped behavioral spaces of each analyzed population and the stereotyped behavioral space of the wild-type population across developmental windows. Different environmental conditions of the same genotype are aggregated for presentation in the heatmap. Color code indicates relative distance between populations (see STAR Methods). (C–H) Stereotyped behavioral spaces of *tph-1* (C), *ceh-33* (D), *cpz-1* (E), *D2005.1* (F), *ceh-6* (G), and *Y116A8C* (H) mutant populations (top) and their corresponding distances to the stereotyped behavioral spaces of the wild-type population (bottom) across development. Shown are the first 12 PCs generated by the PCA in each behavioral space. Analyses were performed on distances from the mid-L1 stage to adulthood (45 developmental windows) (see STAR Methods).

Long-term patterns of behavior across development may be modified by the internal state of the individual, as well as by its past or current environment. To ask whether our method could identify consistent and time-specific behavioral plasticity under various internal and external contexts, we repeated the unsupervised analysis in 30 additional populations, subjected to multiple neuronal and environmental perturbations. The complete dataset (a total of 2,199 individuals across 31 populations) includes populations mutant for various neuronally expressed genes,^19^^,^^20^ as well as neuromodulatory mutants and environmentally perturbed populations (early-life starvation). The quantification of average distances between stereotyped behavioral spaces across all analyzed populations (Figures 5A and 5B) identified effects on stereotyped behavioral patterns during development. As expected, we found that *tph-1* mutant populations, which are deficient for serotonin production, showed a large distance from the wild-type population (average distance: 0.24) (Figures 5B and 5C), as well from all other analyzed populations (Figure 5A) across many developmental windows. These behavioral differences of serotonin-deficient individuals reflect underlying stereotyped PC modes of fast curvature changes (Figure 5C), recapturing their high roaming activity across developmental stages that was previously identified in *tph-1* individuals using pre-defined parameters.^16^^,^^21^ In addition, we found that populations that were exposed to early-life starvation across different genotypes clustered together, suggesting common modes of behavioral responses to early stress across development (Figure 5A).^17^ Interestingly, we found that a fraction of the populations that are mutant for neuronally expressed genes^19^^,^^20^ showed both homogeneous and time-specific effects on trajectories of stereotyped behavioral spaces across development (Figures 5B and 5D–5H). For instance, animals mutant for the homeobox gene *ceh-33*^22^ or for the *cpz-1* enzyme^23^ showed substantial differences in behavioral spaces across most of the developmental stages (L1–L4) (average distance: 0.20 and 0.17, respectively), relative to the wild-type population (Figures 5B, 5D, and 5E). However, these distances were more pronounced in the first half of the L2, L3, and L4 stages, indicating temporal regulation within developmental stages. Additionally, we identified more time-limited alterations in stereotyped behavioral spaces, such as in the stereotyped patterns of individuals mutant for the neuronally expressed homeobox gene *ceh-6*^24^ and for the D2005.1 gene, which is predicted to function in the adenosine-to-inosine (A-I) RNA editing process^25^ and showed a larger difference during the L1 and L2 stages, compared to later developmental stages (average distance: 0.14 and 0.16, respectively) (Figures 5B, 5F, and 5G). These results suggest that neuronally expressed genes with diverse functions, such as genes that are involved in cell differentiation processes and other regulatory pathways, may be involved in shaping long-term behavioral structures across development. In contrast, a fraction of the analyzed populations, such as mutants for the predicted gene Y116A8C.19, showed relatively minor differences in stereotyped behavioral spaces across development, relative to the wild-type population (average distance: 0.12) (Figures 5B and 5H). Overall, these findings show both consistent and stage-specific plasticity of developmental trajectories of stereotyped behavioral spaces, identified by unsupervised behavioral classification across multiple populations.

### Identification of temporal patterns of long-term behavioral uniqueness across conditions

**Figure 6:**
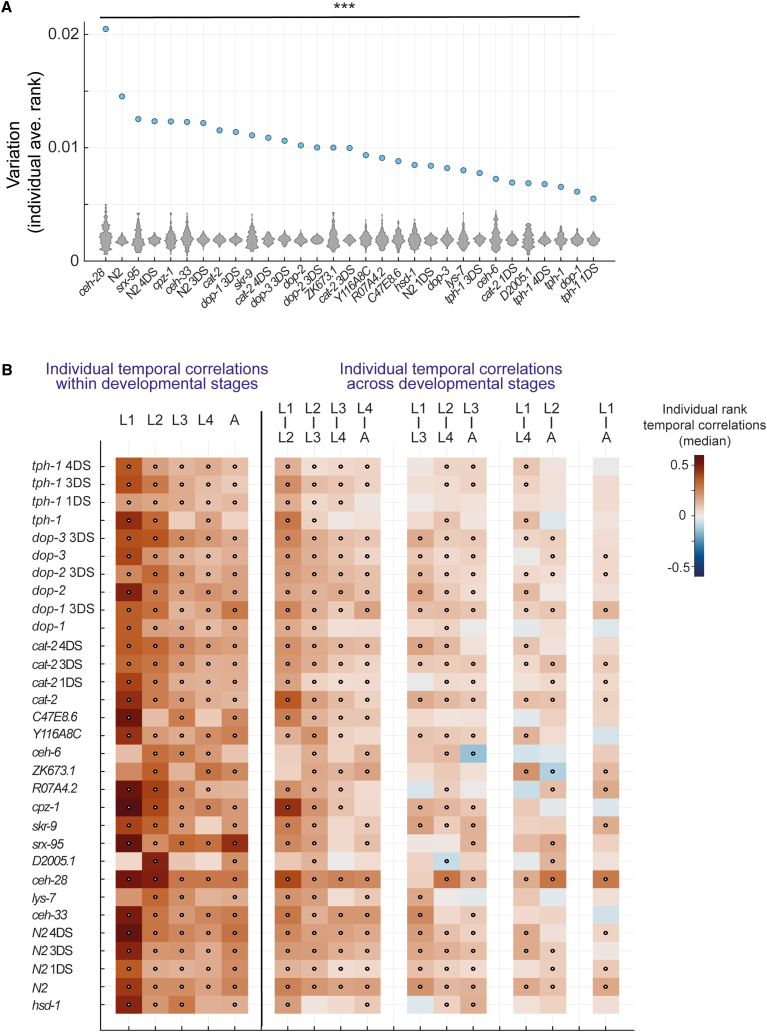
Plasticity in long-term individuality patterns of behavioral uniqueness across populations (A) Variation in average relative rank of behavioral space uniqueness across developmental windows among individuals within all analyzed populations (blue dots) compared to variation among individuals generated from a shuffled rank dataset (distributions of 1,000 runs, gray). ^∗∗∗^*p* < 0.001 (false discovery rate [FDR] corrected) by bootstrap analysis (see STAR Methods). (B) Heatmap represents median temporal correlations (Pearson correlation) between individual uniqueness rank across all pairs of developmental windows in all analyzed populations. Dots represent the significance of median temporal correlations (*p* < 0.05, FDR corrected) by bootstrap analysis, compared to a shuffled rank dataset of the same population. Analyses include individuals with a full trajectory of PCA spaces from the mid-L1 stage to adulthood (45 developmental windows) (see STAR Methods).

To have an extended view, using the developmental behavioral atlas, of how long-term individuality patterns are reshaped across different neuromodulatory and environmental contexts, we quantified the unique behavioral spaces of all individuals within the analyzed populations. Similar to the wild-type population (Figures 4 and S4), we analyzed the relative distance of each single individual to the stereotyped behavioral spaces of its own population and quantified temporal correlations between behavioral uniqueness ranks of all individuals across and within developmental windows (Figure S5B). These analyses allowed us to expose the diversity in temporal patterns of consistency in behavioral uniqueness that may arise homogenously across development time or during specific developmental periods. By initially analyzing the distributions of total temporal correlations between ranks of individual uniqueness across all developmental windows, we found significant individual consistency in behavioral uniqueness levels within all analyzed populations, relative to a population-matched shuffled dataset (Figure S5B). Similarly, quantifying the variation in average behavioral uniqueness rank across development within the different populations showed that while variation levels are diverse, all analyzed populations had extreme individual biases in behavioral uniqueness levels across development, compared to a population-matched shuffled dataset (Figure 6A). These results show that persistent individuality in the uniqueness levels of posture dynamics modes is widespread and can be efficiently detected using the unsupervised inference of individual-specific behavioral spaces within multiple populations.

To further ask whether individuality patterns may be non-homogenously organized during development time in the different populations, we separately analyzed the temporal correlations between behavioral uniqueness ranks of individuals within and between specific developmental stages (Figure 6B). We found that multiple populations showed non-homogenous behavioral consistency, which changed across different developmental periods (Figure 6B). For example, we found that animals mutant for the dopamine receptor DOP-1^26^ that is expressed in a limited number of dopamine-sensing neurons, had non-significant consistency in behavioral uniqueness levels between the adult stage and all other stages (L1–L4) as compared to higher and significant consistency in behavioral uniqueness rank within the adult stage and within all other developmental stages (Figures 6B and S6A). These patterns of temporal correlations were not shown in 3-day starved *dop-1* individuals that had relatively homogeneous consistency within and across all developmental stages (Figures 6B and S6B), suggesting an effect of the early-life environment on the temporal organization of long-term individuality patterns of posture dynamics. Furthermore, we also found specific temporally non-homogenous alterations of behavioral consistency in mutants for neuronal genes whose effect on behavior has not been studied before. For instance, individuals mutant for the gene D2005.1 (predicted to be involved in A-to-I editing)^25^ showed significant consistency in uniqueness levels of behavioral spaces that is higher within the L2 stage (Figures 6B and S6C), and mutants for the Homeobox gene *ceh-6*^24^ did not show significant long-term temporal correlations between the L1 stage and all other developmental stages, as well as within the L1 stage (Figures 6B and S6D).

In summary, these results show the organization of consistent behavioral uniqueness across populations subjected to various neuronal and external perturbations and demonstrate the power of unsupervised analysis of individual-unique behavioral spaces in exposing diverse temporal structures of long-term individuality.

## Discussion

Behavioral structures are highly dynamic throughout development time, representing a mix of stereotyped patterns that are shared by many individuals within the population and unique behavioral modes that are specific to the individual. In this work, we generated a complete and continuous developmental atlas of posture dynamics during the spontaneous behavior of *C. elegans* and used unsupervised inference of underlying behavioral modes for studying the organization of stereotyped and individual-unique behavioral spaces during development time. Across species, underlying behavioral states had been previously characterized at specific developmental periods by temporal body-posture changes, which reflect the movement of the animal in space.^1^^,^^2^^,^^3^^,^^4^^,^^5^^,^^6^^,^^7^^,^^8^^,^^9^^,^^27^ However, how the spectrum of posture dynamics modes continuously progresses throughout the complete developmental trajectory within and across all stages and whether different individuals explore unique trajectories of posture dynamics as they develop is not clear.

Behavioral patterns along the complete developmental trajectory were previously studied using pre-defined locomotory parameters that are based only on the individual’s position, such as the fraction of time that an animal roams or the speed of movement.^16^^,^^17^ While these behavioral analyses identified multiple neuromodulatory and environmental effects on stage-specific behavioral patterns across development, the behavioral characterization using pre-defined parameters represents a limited view of the full developmental progression of behavioral spaces. Here, we combined continuous extraction of the animal’s posture across a full development time and unsupervised inference of low-dimensional behavioral modes to define the complete developmental trajectory of posture dynamics spaces in ∼2,200 individuals within tens of *C. elegans* populations. In particular, we constructed a stereotyped behavioral space in each developmental window that was defined by its dimensions that represent the dominant movement modes within the population, such that posture dynamics patterns of all individuals could be then represented as points within this behavioral space. This approach allowed us to study temporal changes in stereotyped behavioral spaces across the complete developmental trajectory.

To unbiasedly detect underlying dominant behavioral modes that define the behavioral spaces, we performed dimensionality reduction (using PCA) on all posture dynamics sequences within the population in each developmental window. This analysis detected significant PC dimensions across development that represent the underlying behavioral modes that are shared by individuals within the population. We further found that the identified sequence of stereotyped behavioral spaces is not fixed in time but rather shows divergence as development progress. Interestingly, the developmental trajectory of behavioral spaces was smooth over time, implying a continuous gradual change in the spectrum of movement patterns. As the *C. elegans* nervous system is being constantly structured and shaped during post-embryonic development, we hypothesized that the continuous and gradual progression of stereotyped behavioral spaces across developmental timescales may reflect underlying temporal maturation of neuronal circuits and their inter-connections.^28^^,^^29^^,^^30^^,^^31^

Individuals within the same population, even when genetically and environmentally matched, show wide behavioral diversity.^10^^,^^11^^,^^12^^,^^13^^,^^14^^,^^15^^,^^16^^,^^17^ To further utilize the long-term unsupervised inference of underlying modes of posture dynamics for identifying inter-individual behavioral variation, we dissected low-dimensional PCA behavioral spaces separately for each single individual within the population, at each developmental time window. We hypothesized that a fraction of the individuals may explore unique behavioral spaces that include rare modes of posture dynamics that are under-represented in the stereotyped behavioral space of the population. Thus, the construction of individual-specific behavioral spaces allows the quantification of the level of behavioral uniqueness of each single individual at each developmental window and the extraction of the real locomotory patterns of unique individuals following their unsupervised identification. By systematically comparing the low-dimensional behavioral spaces of each single wild-type individual to the stereotyped behavioral spaces of the population, we found that in each developmental window we could identify highly unique individuals as well as highly stereotypical individuals that are similar to the population. These results show wide individual variation in posture dynamics modes, captured during distinct time windows across the developmental trajectory. Interestingly, we further found that individuals within the wild-type population showed long-term consistency in their uniqueness levels of PCA behavioral spaces across and within all developmental stages, implying that long-term behavioral individuality across development can be broadly characterized by unsupervised analysis of posture dynamics. While underlying differences among isogenic individuals may include variation in the nervous system structure,^29^^,^^32^^,^^33^^,^^34^ as well as inter-individual differences in gene expression and neuromodulation,^35^^,^^36^^,^^37^^,^^38^ further studies are required to link these underlying variations to long-term behavioral diversity across developmental timescales.

Following the unsupervised detection of stereotyped and individual-specific behavioral spaces within populations, we sought to study the plasticity of long-term variation in behavior by analyzing multiple mutant populations for neuromodulatory genes that were studied before using pre-defined parameters,^16^^,^^17^ various neuronally expressed genes,^19^^,^^20^ and environmentally perturbed populations (early life stress).^17^ These analyses showed temporally non-homogenous effects on both the stereotyped trajectories of behavior across development and the time distribution of behavioral consistency of individuals. In particular, this approach recaptured previously known effects on behavior (e.g., in the *tph-1* serotonin-deficient populations),^16^^,^^21^ as well as characterized the behavioral effects of multiple neuronally expressed genes. The identified behavioral effects of these neuronally expressed genes permits the further investigation of their specific function within the nervous system in controlling temporal and inter-individual variation in behavior. More generally, as long-term patterns of behavior during development are tightly structured across different species,^37^^,^^39^^,^^40^^,^^41^ we suggest that a combination of neuronal and environmental effects may constrain the “landscape” of possible individual trajectories across development to shape variation within the population.

While the methods in this study were developed and used for extracting developmental trajectories of stereotyped and unique behavioral spaces in *C. elegans*, similar approaches may be used to classify long-term behavioral variation also in other organisms in which posture dynamics can be continuously and efficiently measured across development. In addition, the available developmental dataset of *C. elegans*’ behavior will allow developing and using complementary approaches for studying long-term behavioral dynamics at the individual and the population level. Overall, these results present the developmental structure of behavioral spaces and a general framework for the unsupervised inference of behavioral diversity within developing populations.

### Limitations of the study

The experimental and computational methods presented in this study for analyzing single individuals in isolation across development are highly efficient in identifying long-term patterns of behavior and individual variation. However, it is not clear how the social environment of single individuals affects these behavioral patterns. In particular, interactions among individuals, both at the sensory and physical levels, can modify underlying neuronal processes and affect individual variation over long timescales. The current approach is limited to analyzing single animals and should be extended in future studies to monitor interacting animals within populations across a complete developmental trajectory. Additionally, while patterns of posture dynamics across multiple individuals can be resolved at high resolution using the behavioral imaging system, the simultaneous monitoring of behavior and neuronal activity is limited using the current approach. In future studies, to investigate inter-individual variation at the level of the activity of neuronal circuits and their contribution to behavioral diversity, the behavioral analysis framework presented in this study could be integrated into experimental systems that allow for the monitoring of neuronal activity patterns across developmental stages.

## Resource availability

### Lead contact

Further information and requests for resources and reagents should be directed to and will be fulfilled by the lead contact, Shay Stern (sstern@technion.ac.il).

### Materials availability

All unique/stable reagents generated in this study are available from the lead contact without restriction.

### Data and code availability

• Data have been deposited at google drive and are publicly available as of the date of publication. Links are listed in the key resources table.
• All original code has been deposited at GitHub and is publicly available as of the date of publication. Links are listed in the key resources table.
• Any additional information required to reanalyze the data reported in this work paper is available from the lead contact upon request.

## Acknowledgments

We thank Nabeel Ganem for mutant strains preparation; Sharon Inberg and Manal Marzuk for assistance with the behavioral experiments; and Steve Flavell, David Scher-Arazi, and the members of our laboratory for comments on the manuscript. Some strains were provided by the Caenorhabditis Genetics Center, which is funded by the NIH Office of Research Infrastructure Programs (P40 OD010440). This work was supported by 10.13039/501100000781European Research Council ERC-2019-STG and the 10.13039/501100003977Israel Science Foundation grant 3035/20.

## Author contributions

Conceptualization, Y.H. and S.S. Methodology, Y.H., R.A.N., and S.S. Software, Y.H. Formal analysis, Y.H., R.A.N., and S.S. Investigation, Y.H., R.A.N., and S.S. Writing – original draft, Y.H. and S.S. Writing – review & editing, Y.H., R.A.N., and S.S. Funding acquisition, S.S.

## Declaration of interests

The authors declare no competing interests.

## STAR★Methods

### Key resources table

**Table:** 

REAGENT or RESOURCE	SOURCE	IDENTIFIER
**Deposited data**		

Behavior dataset	This study	https://drive.google.com/drive/folders/1QsM1kckYJIkfQrgasI6YyeikE5YIMALB?usp=sharing

**Experimental models: Organisms/strains**		

Wild-type Bristol N2	N/A	N/A
*tph-1(mg280)* II	H.R. Horvitz lab, MIT	MT15434
*hsd-1(mg433)* I	CGC	GR2063
*ceh-33(ok1362)* V	CGC	RB1271
*lys-7(ok1385)* V	CGC	RB1286
*ceh-28(ok1833)* X	CGC	RB1528
*D2005.1(ok2689)* I	CGC	RB2031
*srx-95(ok3399)* II	CGC	RB2460
*skr-9(ok3453)* IV	CGC	RB2493
*cpz-1*(ok497) I	CGC	RB732
*R07A4.2(sts01)* X	This study	SST001
*ZK673.1*(*sts02*) II	This study	SST005
*ceh-6*(gk679) I	CGC	VC1481
*Y116A8C.19* (gk958) IV	CGC	VC2052
*C47E8.6*(gk1232) V	CGC	VC2343
*cat-2* (e1112) II	CGC	CB1112
*dop-1* (vs100) X	CGC	LX645
*dop-2* (vs105) V	CGC	LX702
*dop-3* (vs106) X	CGC	LX703

**Software and algorithms**		

FlyCapture	FLIR	https://www.flir.com/
MATLAB	Mathworks	https://www.mathworks.com
Julia		https://julialang.org/
Analysis scripts	This study	https://github.com/yha/Elegans https://doi.org/10.5281/zenodo.13255237 https://github.com/yha/ElegansTimeSeries https://doi.org/10.5281/zenodo.13255184

**Other**		

12 MP USB3 Flea camera	FLIR	Cat#FL3-U3-120S3C-C
LED backlights	Metaphase Technologies	Cat#99021169
Temperature control (cooling unit- Peltier element)	TE technology	Cat#AC-027

### Experimental model and study participant details

#### *C. elegans* populations

*C. elegans* strains used in this study: Wild-type Bristol N2 (*n* = 123, 1DS *n* = 99, 3DS *n* = 119, 4DS *n* = 115); MT15434 *tph-1*(mg280) II (*n* = 51, 1DS *n* = 87, 3DS *n* = 96, 4DS *n* = 104); GR2063 *hsd-1*(mg433) I (*n* = 23); RB1271 *ceh-33*(ok1362) V (*n* = 29); RB1286 *lys-7*(ok1385) V (*n* = 29); RB1528 *ceh-28*(ok1833) X (*n* = 17); RB2031 *D2005.1*(ok2689) I (*n* = 25); RB2460 *srx-95*(ok3399) II (*n* = 19); RB2493 *skr-9*(ok3453) IV (*n* = 24); RB732 *cpz-1*(ok497) I (*n* = 28); SST001 *R07A4.2*(*sts01*) X (*n* = 28); SST005 *ZK673.1*(*sts02*) II (*n* = 36); VC1481 *ceh-6*(gk679) I (*n* = 24); VC2052 *Y116A8C.19* (gk958) IV (*n* = 32); VC2343 *C47E8.6*(gk1232) V (*n* = 23); CB1112 *cat-2* (e1112) II (*n* = 124, 1DS *n* = 98, 3DS *n* = 124, 4DS *n* = 85); LX645 *dop-1* (vs100) X (*n* = 73, 3DS *n* = 133); LX702 *dop-2* (vs105) V (*n* = 111, 3DS *n* = 143); LX703 *dop-3* (vs106) X (*n* = 82, 3DS *n* = 95). ‘DS’ indicate days of starvation.

### Method details

#### Growth conditions

*C. elegans* worms were maintained on NGM agar plates, supplemented with E. coli OP50 bacteria as a food source. For behavioral tracking, we imaged single individuals grown in custom-made laser-cut multi-well plates. Each well (10mm diameter) was seeded with a specified amount of OP50 bacteria (10 μL of 1.5 OD) that was UV-killed before the experiment to prevent bacterial growth. For the starvation experiments, eggs were collected from isogenic populations using a standard bleaching protocol, into an agar plate without OP50 bacteria. Newly hatched L1 larvae were starved for a specified time window (L1 arrest of 1, 3 or 4 days) before being transferred to the imaging multi-well plates.

#### Imaging system

Longitudinal behavioral imaging was performed using custom-made imaging systems. Each imaging system consists of an array of six 12 MP USB3 cameras (Pointgrey, Flea3) and 35 mm high-resolution objectives (Edmund optics) mounted on optical construction rails (Thorlabs). Each camera images up to six wells, each containing an individual grown in isolation. Movies are captured at 3 fps with a spatial resolution of ∼9.5 μm. For uniform illumination of the imaging plates we used identical LED backlights (Metaphase Technologies) and polarization sheets. To tightly control the environmental parameters during the experiment, imaging was conducted inside a custom-made environmental chamber in which temperature was controlled using a Peltier element (TE technologies, temperature fluctuations in the range of 22.5 ± 0.1°C). Humidity was held in the range of 50% +/− 5% with a sterile water reservoir and outside illumination was blocked, keeping the internal LED backlights as the only illumination source. Movies from the cameras were captured using commercial software (FlyCapture, FLIR) and saved on two computers (3 cameras per computer; each computer has at least 8-core Intel i7/i9 processor and 64 GB RAM).

### Quantification and statistical analysis

#### Imaging data processing

##### Extraction of trajectories of individuals’ center of mass

To extract behavioral trajectories of each individual’s center of mass across the experiment, captured videos were analyzed by a custom-made script programmed in MATLAB (Mathworks, version 2019b) (Stern et al. 2017). In each video frame and for each behavioral arena, the worm is automatically detected as a moving object by background subtraction, and the coordinates of its visual center of mass are logged. In each experiment, 600,000–700,000 frames per individual are analyzed using ∼50 processor cores in parallel to reconstruct the full behavioral trajectory of individuals over days of measurements across development. The total time of image processing was 3–7 days per experiment (tens of individuals across development). Egg hatching time of each individual in the experiment is automatically marked by the time when activity can be detected in the behavioral arena. The middle of the lethargus periods, in which animals stop their locomotion and molt, were manually marked as the transition points between different stages of development (based on 10s-time-scale speed trajectories, smoothed over 300 frames). To synchronize temporal behavioral trajectories of different individuals we age-normalized individuals by dividing the behavioral trajectory of each life stage into a fixed number of time bins.

#### Posture analysis and head-tail detection across development

To find the worm’s midline in each frame, the worm’s contours were first extracted from cropped background-subtracted images (example videos are included in the behavior dataset) at a fixed grayscale level threshold, using the Marching Squares algorithm as implemented in the Julia package *Contour.jl*.^42^ If multiple contours are found, the one that consists of most points is selected. This yields a list $p_{i},\left(i=1,..k\right)$ of k points $p_{i}=\left(x_{i},y_{i}\right)$ around the worm’s contour in each frame where at least one contour was detected. In each of these frames, the two ends of the worm were identified as peaks of curvature along the contour as follows: First, the contour was smoothed by circularly convolving the list of points with a Gaussian kernel of width $\sigma_{c}=2$. Curvature of the smoothed contour ${\left({\tilde{p}}_{i}\right)}_{i=1}^{k}$ was estimated at each contour point as the change in angle at the point when traversing the contour counter-clockwise, divided by the distance $\frac{1}{2}\left|{\tilde{p}}_{i+1}-{\tilde{p}}_{i-1}\right|$, yielding curvature values $\kappa_{i}$. Finally, peaks of curvature, where $\kappa_{i}>\kappa_{i-1}$ and $\kappa_{i}>\kappa_{i+1}$ were identified, and the two with the highest curvature values were selected as the worm’s ends. This results in two coordinates of the worm’s ends in each frame where contour detection was successful.

Next, the two ends are aligned across different frames so that their identity is preserved within each continuous range of successful frames. In each successful frame, the distance traveled by each end since the previous successful frame is computed, and the labels of the two ends are swapped if the swap results in a lower sum of the two distances. The distance ratio, which is the ratio of the sum of distances compared to the sum if the ends are swapped is recorded for each frame. Note that after possibly swapping the labels, the distance ratio is always in the range [0,1].

#### Head and tail detection

To identify which of the two ends is the head, we first omit frames where the continuous tracking of each end is less certain. Frames are skipped if they satisfy at least one of four conditions: (1) more than one contour was detected, (2) the distance ratio exceeded 0.2, (3) at least two frames in a row where no distance ratio could be computed (either no contour was detected or some failure occurred in the procedure described above, such as only one peak in contour curvature) or (4) the contour was too round. Roundness (criterion (4)) was computed as the ratio of the area of the contour’s interior to its length, and *Z* score of this roundness parameter was computed for each frame i relative to roundness values in frames i−5000 to i+5000. Frames where the roundness *Z* score exceeded 3 were skipped. The roundness criterion was employed to avoid frames where a wrong contour is detected for a curled-up worm.

By omitting these frames, time is divided into segments consisting of consecutives non-omitted frames. We classify which of the two ends is the head, separately in each time segment. Identification of the head is based on its faster side-to-side movements, compared to the tail, which can be detected during both roaming and dwelling. The trajectories of each end are first smoothed by convolution with a Gaussian kernel of width σ=5, to obtained two smoothed series of points $e_{i}^{\left(1\right)},e_{i}^{\left(2\right)}$. The speed at each end is estimated as $s_{i}^{\left(j\right)}=\frac{1}{2}\left|e_{i+1}^{\left(j\right)}-e_{i-1}^{\left(j\right)}\right|$, and the log speed ratio $\text{ls}r_{i}=\log\left(s_{i}^{\left(1\right)}/s_{i}^{\left(2\right)}\right)$ is computed at each frame. End 1 is selected as the head if the average log speed ratio across the time segment is positive, otherwise end 2 is selected as the head. The absolute value of the average log speed ratio is recorded as a confidence measure for the head identification in the time segment. All subsequent analysis was restricted to segments where the confidence exceeded 0.05.

#### Midline identification and curvature analysis

Following head and tail identification, each contour was split into two parts at the head and tail, and an approximately length-parameterized cubic spline was fitted to each part, following the direction from the head to the tail. Splines were fitted using the Julia package *Dierckx.jl*,^43^ which is a wrapper around the Fortran library *dierckx (Dierckx, 1993)*. Midlines were then computed by sampling each of the two splines at a set of 41 equally spaced spline parameter values (s=0,0.025,0.05,…,1), averaging each pair of samples to obtain points along the worm’s midline, and fitting a spline through these points. This spline was iteratively resampled at the same values of s until the resulting points were approximately equally spaced. The result of the last iteration is 41 equally spaced points along the worm’s midline.

The angle between each three consecutive points was computed, yielding an estimate of the worm’s midline curvature at each of 39 points. Due to higher levels of noise in the procedure near the ends of the worms, we removed the two extreme points, obtaining a representation of the midline as a vector of k=37 curvature values. We collected curvature vectors at each successful frame for each individual, yielding a matrix representation of the worm’s posture dynamics over development time.

#### Posture dynamics PCA

To characterize dominant posture dynamics modes across development throughout the population, we first aligned the developmental time of all individuals, dividing each larval stage into 10 time bins, and analyzed the posture data in each developmental time bin separately. In each bin, we performed PCA on the pool of all overlapping 10-s curvature matrices from the entire population, from each window contained in a time segment where head detection passed the confidence threshold (see “Head detection” above). PCA was fitted to the data using the Julia package *MultivariateStats.jl*. PCA analysis yields, for each time bin, characteristic modes (PC vectors) of curvature dynamics whose linear combinations best reconstruct curvature matrices, and corresponding variance values, describing how much of the variance in curvature matrices is explained by each PC. This analysis was repeated for each analyzed population, as well as for each individual worm separately.

To choose the number of PCs used in each time bin, we estimated PCA reconstruction error for different dimensionalities, using the cross-validation method.^44^ Dimensionality estimation was performed for each of the analyzed populations in each time bin. For individual worms, the dimensionality used was that obtained from the population estimate, except when fewer dimensions were sufficient to explain 99% of observed variance in the individual, in which case only those dimensions were used. The dimensionality chosen in each time bin for each population was the first value of d where the base-10 logarithm of reconstruction error from d−1 dimensions decreased by less than 0.01 when increasing the dimensionality to d. For computational tractability, in each population and time bin, N=1000 time windows were sampled for use in the dimensionality estimation procedure. PCA is performed on the pool of curvature matrices for these time windows, represented as a Tk×N matrix M where each column is a T×k curvature matrix laid out as a vector. For cross-validation, a random set of rows and columns is chosen to be held out as a validation set. We mark by $\hat{M}$ a rearrangement of M’s rows and columns where the held-out rows and columns appear first, so that $\hat{M}$ may be decomposed as $\hat{M}=\left(\begin{array}{cc} A & B\\ C & D \end{array}\right)$, where A corresponds to the held-out rows and columns. Each row and column of M is chosen to be held out independently with probability $\sqrt{0.1}$, so that A contains, on average, 10% of M’s entries. PCA is then applied to D, yielding a decomposition $D\approx U_{D}V_{D}^{T}$ to principal axes in the column of $U_{D}$ and PCA scores in $V_{D}$. These are used to reconstruct scores ${\hat{V}}_{C}$ for C as the least squares solution of $C\approx U_{D}{\hat{V}}_{C}^{T}$ and principal axes ${\hat{U}}_{B}$ for B as the least squares solution of $B\approx {\hat{U}}_{B}V_{D}^{T}$. An estimate of A from d dimension is then obtained as ${\hat{A}}^{\left(d\right)}={\hat{U}}_{B}^{\left(d\right)}{{\hat{V}}_{C}^{\left(d\right)}}^{T}$, where ${\hat{U}}_{B}^{\left(d\right)}$ and ${\hat{V}}_{C}^{\left(d\right)}$ are the first d columns of ${\hat{U}}_{B}$ and ${\hat{V}}_{C}$ respectively, and a relative reconstruction error computed as $\varepsilon_{d}≔\sum_{i,j}{\left(A_{ij}-{\hat{A}}_{ij}^{\left(d\right)}\right)}^{2}/\sum_{i,j}{\left(A_{ij}-\bar{A}\right)}^{2}$, where $\bar{A}$ is the mean of all elements in A.

For each time bin in each population, we repeated this procedure k=10 times for each candidate dimensionality d in the range 1:50, to obtain k reconstruction error estimates per choice of dimensionality d. For each dimensionality, we obtain several bootstrap estimates of the mean reconstruction error by resampling k error estimates with replacement from the set of k estimates, to obtain K=10,000 estimates of the mean error. Next, we estimate the first value of dimensionality d where the base-10 logarithm of mean reconstruction error decreases by less than 0.01 when increasing the dimensionality by 1. This value is estimated K times, once from each resampling of the k error estimates, and finally averaged and rounded to the nearest integer to obtain the dimensionality estimate.

#### Comparison of PCAs

To assess differences across individual-unique PCA spaces and population PCA spaces, in each developmental time bin, we quantify the (unnormalized) distance of the population or individual PCA (V) to the reference PCA (W), where each PCA is truncated to the number of significant PCs determined by dimensionality estimation. Specifically, to compare significant PCs (eigenvectors) $v_{1},\ldots ,v_{d}$ with associated variances (eigenvalues) $\lambda_{1},\ldots \lambda_{d}$ to reference PCs $w_{1},\ldots ,w_{r}$ (where d,r are the estimated dimensionalities of the PCAs), we compute the expected squared distance of a random vector v to the subspace spanned by reference PCs $w_{1},\ldots ,w_{r}$, where v is sampled from the distribution induced by the significant PCs of V and their associated variances: namely, the distribution of Vz where V is the projection matrix with columns $v_{1},\ldots ,v_{d}$ and z is a d-dimensional vector with independent components of variances $\lambda_{1},\ldots \lambda_{d}$. The distance of an arbitrary vector v from the subspace spanned by the reference PCs is

$$\left|v-\sum_{j=1}^{r}\left(v\cdot w_{j}\right)w_{j}\right|=\left|\left(I-WW^{T}\right)v\right|\text{,}$$

where W is a projection matrix with columns $w_{1},\ldots ,w_{r}$. Therefore, the required expected square distance is given by

$$\tilde{d}\left(V,W\right)≔E\left[{\left|\left(I-{WW}^{T}\right)Vz\right|}^{2}\;\right]=E\;\text{tr}\left[\left(I-{WW}^{T}\right)Vzz^{T}V^{T}\left(I-WW^{T}\right)\right]=\text{tr}\left[\left(I-{WW}^{T}\right)V\;\text{diag}\left(\lambda \right)V^{T}\left(I-WW^{T}\right)\right]=\sum_{i=1}^{d}{\left|\left(I-WW^{T}\right)v_{i}\sqrt{\lambda_{i}}\right|}^{2}$$

i.e., the sum of squared distances of loading vectors $\surd \lambda_{i}v_{i}$ to the subspace of W.

This distance is then normalized by the total variance explained by significant PCs of V to obtain the *relative distance* measure in the range [0,1] as

(Equation 1) $$d\left(V,W\right)≔\frac{\tilde{d}\left(V,W\right)}{\sum_{i=1}^{d}\lambda_{i}}=\frac{\sum_{i=1}^{d}{\left|\left(I-WW^{T}\right)v_{i}\right|}^{2}\lambda_{i}}{\sum_{i=1}^{d}\lambda_{i}}$$

For comparisons between populations, or between different time bins of the same population, we used the symmetrized distance $\frac{1}{2}\left(d\left(V,W\right)+d\left(W,V\right)\right)$, with the number of dimensions used chosen according to dimensionality estimation procedure described above (see *Dimensionality Estimation*). For comparing individuals to their population, we took the individual’s PCA as V, and the population PCA as the reference W in Equation 1. For the dimensionality of the individual’s PCA in each time bin (value of d in Equation 1, we used the dimensionality estimated from the population, except where fitting the individual’s PCA (up to 99% explained variance) resulted in fewer PCs than the population estimate, in which case all the individual’s PCs were used. For comparing individuals to other individuals, or between developmental time bins of the same individuals, we used the symmetrized distance $\frac{1}{2}\left(d\left(V,W\right)+d\left(W,V\right)\right)$, with the dimensionality chosen as described above for comparison of individuals to the population.

#### PCA distance by temporal difference

To verify that the PCA distance measure captures aspects of behavior that vary smoothly at the timescale chosen for the analysis time bins, we assessed the dependence of PCA distance between time bins on the temporal distance. For each temporal distance, we averaged the PCA distance in the wild type population of all pair of bins at that distance from each other. We computed Pearson correlation between these averages and the temporal distance. Statistical significance of the observed correlation was tested against the Pearson correlations obtained from 1000 shuffles of the PCA distances, where the time bins were randomly shuffled.

#### Long-term consistency analysis of PCA space uniqueness

To analyze long-term consistency in relative behavioral uniqueness levels, we ranked all individuals based on their relative distance to the population (1). As the ranking required a complete individual-to-individual comparison in each developmental window, we only included individuals that had a full trajectory of PCA spaces across developmental time bins 6 to 50 (mid-L1 to adulthood), due to relatively increased rate of missing PCAs during earlier time bins (N2 (*n* = 112, 1DS *n* = 95, 3DS *n* = 112, 4DS *n* = 108); *tph-1* (*n* = 44, 1DS *n* = 78, 3DS *n* = 92, 4DS *n* = 91); *cat-2* (*n* = 114, 1DS *n* = 83, 3DS *n* = 114, 4DS *n* = 79); *hsd-1* (*n* = 21); *ceh-33* (*n* = 17); *lys-7* (*n* = 20); *ceh-28* (*n* = 10); *D2005.1* (*n* = 18); *srx-95* (*n* = 13); *skr-9* (*n* = 22); *cpz-1* (*n* = 17); *R07A4.2* (*n* = 23); *ZK673.1* (*n* = 17); *ceh-6* (*n* = 12); *Y116A8C.19* (*n* = 30); *C47E8.6* (*n* = 20); *dop-1* (*n* = 72, 3DS *n* = 124); *dop-2* (*n* = 105, 3DS *n* = 127); *dop-3* (*n* = 78, 3DS *n* = 89)). In each time bin, individuals were ranked within each experiment by their relative distance to the population. Ties were resolved as fractional ranks (“1 2.5 2.5 4 ranking”). This produces a rank $r_{i,k}$ for the i th individual in the k th time bin, between 1 and $n_{i}$, where $n_{i}$ is the number of individuals measured in the experiment which includes individual i. These ranks were normalized to obtain *relative uniqueness rank* values between 0 and 1, as $u_{i,k}=\left(r_{i,k}-\frac{1}{2}\right)/n_{i}$. Thus, a relative uniqueness rank $u_{i,k}=\frac{1}{2}$ is obtained when the relative distance of worm i in bin k to the population is the median distance for that experiment. A higher relative rank occurs in a time bin where a worm’s PCA is more unique, in the sense of a larger relative distance to the population than other worms in its experiment, and a lower relative rank occur where the worm’s PCA is more stereotypical. Particularly, in each time bin, the worms with the highest and lowest relative distance to the population are assigned relative ranks (1−1/(2n)) and 1/(2n), respectively, where n is the number of worms in the experiment.

#### Distances in shared PCA space

As a complementary approach distances between individuals and their population were also computed from their representation in a shared PCA space. This analysis was performed using the population’s PCA computed at each time bin, as well as using a shared PCA across all time bins. To equally weigh all developmental time bins, the shared PCA was computed from a sample of 10,000 time windows from the population in each of the 50 time bins, for a total of 500,000 data points.

We computed each individual’s PCA scores for each time window, and the variances of these scores were computed in each developmental time bin. For the shared PCA across all time bins, we also computed variance of PCA scores across the time windows sampled from the population in each time bin.

From these variances, fraction of variance explained by PC k for individual i in bin b is computed as

$$f_{i,k,b}=\frac{\sigma_{i,k,b}^{2}}{\sigma_{i,b}^{2}}$$

where $\sigma_{i,k,b}^{2}$ is the variance of the k-th PC score for individual i in bin b, and $\sigma_{i,b}^{2}$ the total variance in the time bin – i.e., the sum of score variances over all PCs, or equivalently, the sum of variances of each component of the curvature matrix across the time bin.

Similarly, variance fractions are computed for the population as

$$f_{\text{pop},k,b}=\frac{\sigma_{\text{pop},k,b}^{2}}{\sigma_{\text{pop},b}^{2}}$$

where $\sigma_{\text{pop},k,b}^{2}$ the variance of the k-th PC score for the population, and $\sigma_{\text{pop},b}^{2}$ the total variance for the population.

These fractional variances were used to compute the distance $d_{i,b}$ of individual i to the population in bin b as

$$d_{i,b}=\sum_{k=1}^{n_{b}}\left|\frac{f_{i,k,b}}{f_{\text{pop},k,b}}-1\right|$$

where $n_{b}$ is the number of dimensions used. When comparing to a separate population PCA at each time bin, $n_{b}$ was chosen by cross validation. For the shared PCA across time bins, $n_{b}$ was chosen to explain 70% of variance in each time bin, i.e., the smallest value such that $\sum_{k=1}^{n_{b}}f_{\text{pop},k,b}>0.7$. Finally, to obtain average ranks of individuals in the shared PCA space, the distances $d_{i,b}$ were ranked within each experiment in each time bin and the ranks normalized to the range (0,1) were averaged across developmental time bins 6–50.

#### Relative uniqueness temporal correlations

We computed correlations of relative uniqueness ranks across individuals between each pair of developmental time bins. To assess statistical significance of these temporal correlation, we compared the median of temporal correlations across all pairs of different time bins in the range 6–50, to medians obtained from 1000 randomly shuffled rank datasets, where ranks in each time bin were shuffled independently within each experiment. The same shuffled datasets were also used to assess significance of the median temporal correlation within each pair of developmental stages separately, and in each of the analyzed populations.

The relative uniqueness rank of each individual was averaged across developmental time bins 6–50 to obtain a global uniqueness measure between 0 and 1, where higher values indicate consistently unique individuals, and lower values indicate consistently stereotypical individuals. To assess statistical significance of individual consistency in relative uniqueness rank across the population, we compared the variance of the mean relative uniqueness rank to those obtained from the same shuffled rank datasets used for significance testing of relative uniqueness temporal correlation. This was repeated in each of the analyzed populations.

#### Clustering of populations

Hierarchical clustering analysis of populations was performed using complete-linkage clustering implemented by the *hclust* function from the Julia package *Clustering.jl*, and based on the symmetrized distance between each pair of populations, averaged across developmental bins 6–50.

#### Lethargus length analysis

To analyze lethargus length, lethargus periods were automatically identified around the manually marked developmental stage boundaries. These periods were identified from the low speed of the worm relative to its speed in the two adjacent developmental stages. First, an estimate $s_{i}=\left|p_{i+3}-p_{i}\right|$ of the worm’s speed was computed at each frame i. Since the videos are at 3 frames per second, this estimate has units of pixels per second.

Analysis proceeded around each manually marked stage boundary $t_{1},\ldots t_{4}$, where $t_{k}$ is the boundary marked between developmental stage k and stage k+1. Individuals where one or more such boundary was marked at a frame where tracking has failed were excluded from lethargus length analysis.

Around each manually marked boundary $t_{k}$, statistics of the speed were collected from frame $a_{k}=0.7\cdot t_{k-1}+0.3\cdot t_{k}$ to frame $b_{k}=0.3\cdot t_{k}+0.7\cdot t_{k+1}$. First, the speed was averaged in consecutive (non-overlapping) windows of 300 frames (100 s) from $a_{k}$ to $b_{k}$. Statistics of these speed averages were collected to obtain their Empirical Cumulative Distribution Function (ECDF) in that range, from which quantile values $q_{i}$ of the average speed are computed for frames in the range $a_{k}\leq i\leq b_{k}$. Any missing quantile value (where worm tracking has failed), was replaced by $\frac{1}{2}$. Quantile values were then smoothed by convolution with a Gaussian kernel of width σ=10 300-frame windows to obtained smoothed quantile values $e_{i}$ at each window. Lethargus period was marked as the largest continuous range of frames around $t_{k}$ where $e_{i}<0.2$. If $e_{t_{k}}\geq 0.2$ lethargus detection was considered to have failed and the worm was excluded from the lethargus length analysis.

Detected lethargus periods were verified manually by observing the speed trace of each worm. One individual was manually excluded from the analysis based on unclear timing of its first lethargus.

Distributions of lethargus lengths were compared using Mann-Whitney test with FDR correction. Statistical significance of lethargus length correlations were computed by comparison to 100,000 shuffles, where worms were shuffled independently for each of the 4 lethargus periods.

#### t-SNE visualizations

A two-dimensional representation of the wild-type population PCA trajectory was generated using t-SNE.^45^ The t-SNE representation was computed using the *TSne.jl* Julia package,^46^ from the symmetrized relative distance $\frac{1}{2}\left(d\left(V,W\right)+d\left(W,V\right)\right)$ between each pair of time bins, with perplexity parameter value 20.

For the t-SNE visualizations of wild-type individuals alongside the wild-type population trajectory, we used PCAs for all individuals and time bins where the output dimension of the fitted PCA was at least 2, as well as PCAs of the wild-type population in all time bins (6157 PCAs in total). Symmetrized relative distances were computed between all pairs of such PCAs, which were used to compute a t-SNE representation with a perplexity parameter value 10.

In all t-SNE visualization, lines representing developmental trajectories were obtained by fitting a cubic spline through the data point.

## Supplemental information

Document S1. Figures S1–S6

Document S2. Article plus supplemental information
