# Supplementary material for: Mapping the developmental structure of stereotyped and individual-unique behavioral spaces in C. elegans
Source: Cell Rep. 2024 Aug 27;43(9):114683. doi: 10.1016/j.celrep.2024.114683 (PMC11422485; doi:10.1016/j.celrep.2024.114683)
Supplement: Document S1. Figures S1–S6 [file mmc1.pdf]

Cell Reports, Volume *43*

Supplemental information

Mapping the developmental structure of stereotyped and individual-unique behavioral spaces in *C. elegans*

Yuval Harel, Reemy Ali Nasser, and Shay Stern

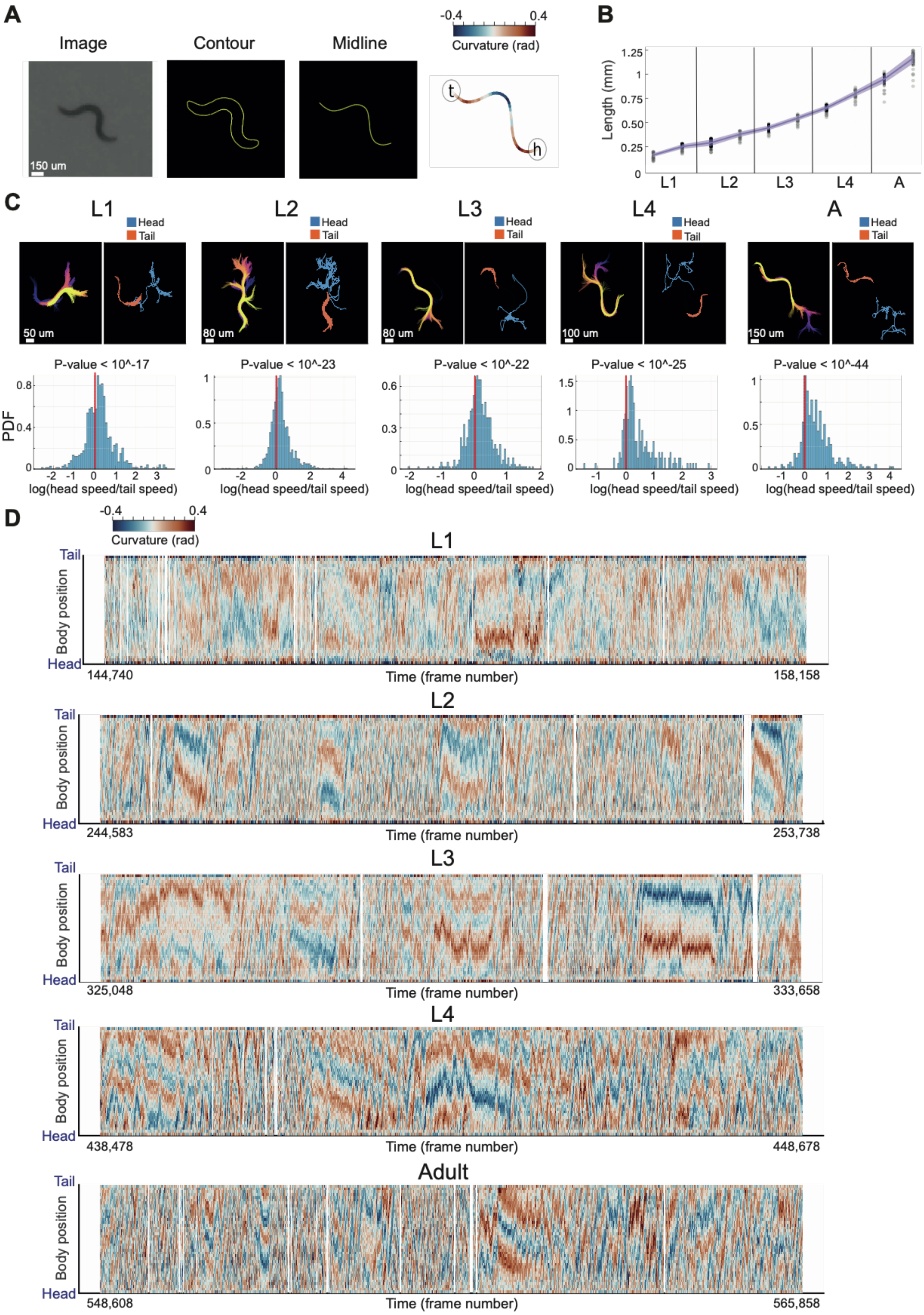
A
Image
Contour
Midline
-0.4
0.4
Curvature (rad)
150 um
t
h
B
Length (mm)
L1
L2
L3
L4
A
C
L1
L2
L3
L4
A
Head
Tail
50 um
80 um
80 um
100 um
150 um
P-value < 10^-17
P-value < 10^-23
P-value < 10^-22
P-value < 10^-25
P-value < 10^-44
PDF
log(head speed/tail speed)
D
Curvature (rad)
Tail
Body position
Head
Time (frame number)
144,740
158,158
244,583
253,738
325,048
333,658
438,478
448,678
Adult
548,608
565,858

Figure S1. Related to Figure 1.

Individual size and posture dynamics quantification across development

(A) An example of the quantification of an individual's contour, body midline, and curvature profile across midline points, homogenously distributed from head to tail ('h' and 't', respectively). Color code marks curvature in each point along the midline. Scale bar is indicated in white. **(B)** Individuals average length (mm) within the wild-type population (n=123) across developmental stages (2 time bins per stage). Each dot represents a single individual. Line indicates average length and shaded area indicated standard error of the mean. **(C)** Head and tail detection across all developmental stages (L1-Adult) based on differences in speed of detected ends of each animal (see Methods). Represented are examples across developmental stages of midline dynamics (top left), smoothed trajectories of detected head (blue) and tail (orange) (top right) and distributions of log(head speed/tail speed) within the time window (bottom). P-value indicates significance of differences in head vs. tail speed (Wilcoxon signed rank test). Color code of midline dynamics marks time within the presented window. Images were enlarged for visual clarity (indicated in white zoom ratio relative to cropped image as in (A)). Scale bars are indicated in white for each developmental stage. **(D)** Examples of continuous midline curvature quantification of an individual across all developmental stages. Shown are time windows that represent 10% of the total time of each developmental stage of the individual (10,000-12,000 sequential frames). Color code marks curvature in each point along the midline. White indicates frames in which midline extraction has failed.

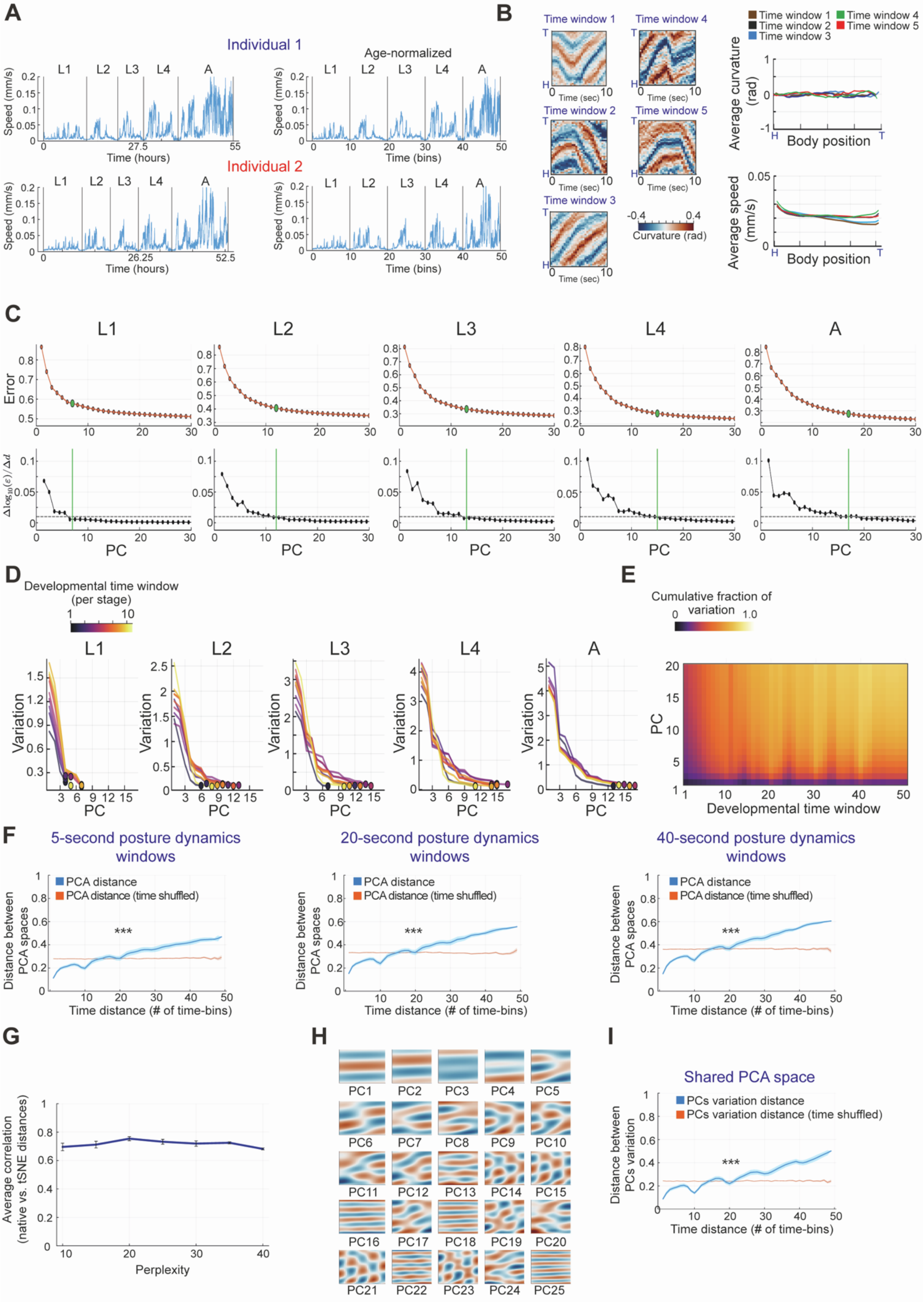
A
Individual 1
Age-normalized
L1 L2 L3 L4 A
Speed (mm/s)
Time (hours)
Time (bins)
Individual 2
B
Time window 1
Time window 2
Time window 3
Time window 4
Time window 5
Time (sec)
Curvature (rad)
Average curvature (rad)
Average speed (mm/s)
Body position
C
Error
PC
D
Developmental time window (per stage)
Variation
E
Cumulative fraction of variation
Developmental time window
F
5-second posture dynamics windows
20-second posture dynamics windows
40-second posture dynamics windows
PCA distance
PCA distance (time shuffled)
Distance between PCA spaces
Time distance (# of time-bins)
G
Average correlation (native vs. tSNE distances)
Perplexity
H
I
Shared PCA space
PCs variation distance
PCs variation distance (time shuffled)
Distance between PCs variation

Figure S2. Related to Figure 2.

Inference and temporal analysis of stereotyped behavioral spaces across developmental windows

(A) Left: Developmental stage classification across development time using low activity lethargus states between stages. Right: Age-normalization for each individual was performed by equally dividing each developmental stage into a fixed number of time bins. Shown are examples of two individuals. **(B)** Examples of five 10-second time windows of posture dynamics (left) and the average curvature and speed across midline points within the time windows (right) (sampled from L3 – 3rd time bin). Color code marks curvature in each point along the midline. **(C)** Examples of dimensionality estimation during specific developmental time windows of wild-type individuals (n=123) across all stages. Shown is the cross-validation relative square error for each choice of dimensionality (top) and the change in the base-10 logarithm of the error with each additional dimension (bottom). Dashed line indicates the threshold of 0.01 for the change in logarithmic error (see Methods). Green line indicates estimated dimensionality. Time windows represented: L1 – 7th time bin, L2 – 7th time bin, L3 – 7th time bin, L4 – 7th time bin, Adult – 5th time bin, out of 10 time bins per developmental stage. **(D)** Variation explained by each PC during all 50 time windows across development. Dots indicate PCA space dimensionality estimate in each of the developmental time windows. Color code marks time window number within each stage (10 windows per developmental stage). **(E)** Heatmap presents the cumulative fraction of variation explained by PCs across developmental time windows. **(F)** Average distance across stereotyped PCA spaces of the wild-type population generated from posture dynamics windows of different sizes (5, 20, or 40-second windows), separated by a specific number of time windows (blue) relative to stereotyped PCA spaces that are shuffled in time (1000 runs, orange). Shaded area represents standard error of the mean. P-value was generated by bootstrapping (see Methods) for the difference in correlation (Pearson) between the real and time-shuffled dataset. **(G)** Average correlation (Pearson) between the distances calculated between the stereotyped PCA spaces at different developmental windows and the distances embedded within a tSNE space using different perplexities (10-40). Error bars represent standard error of the mean (50 random seeds). **(H)** Shown are PC1-PC25 generated by constructing a shared PCA space from all posture dynamics windows across development (fraction of variation explained - 70%). **(I)**

Same as (F) for distances quantified between the PCs fraction of variation explained within a shared PCA space constructed from all posture dynamics windows across development (see Methods). *** P-value<0.001.

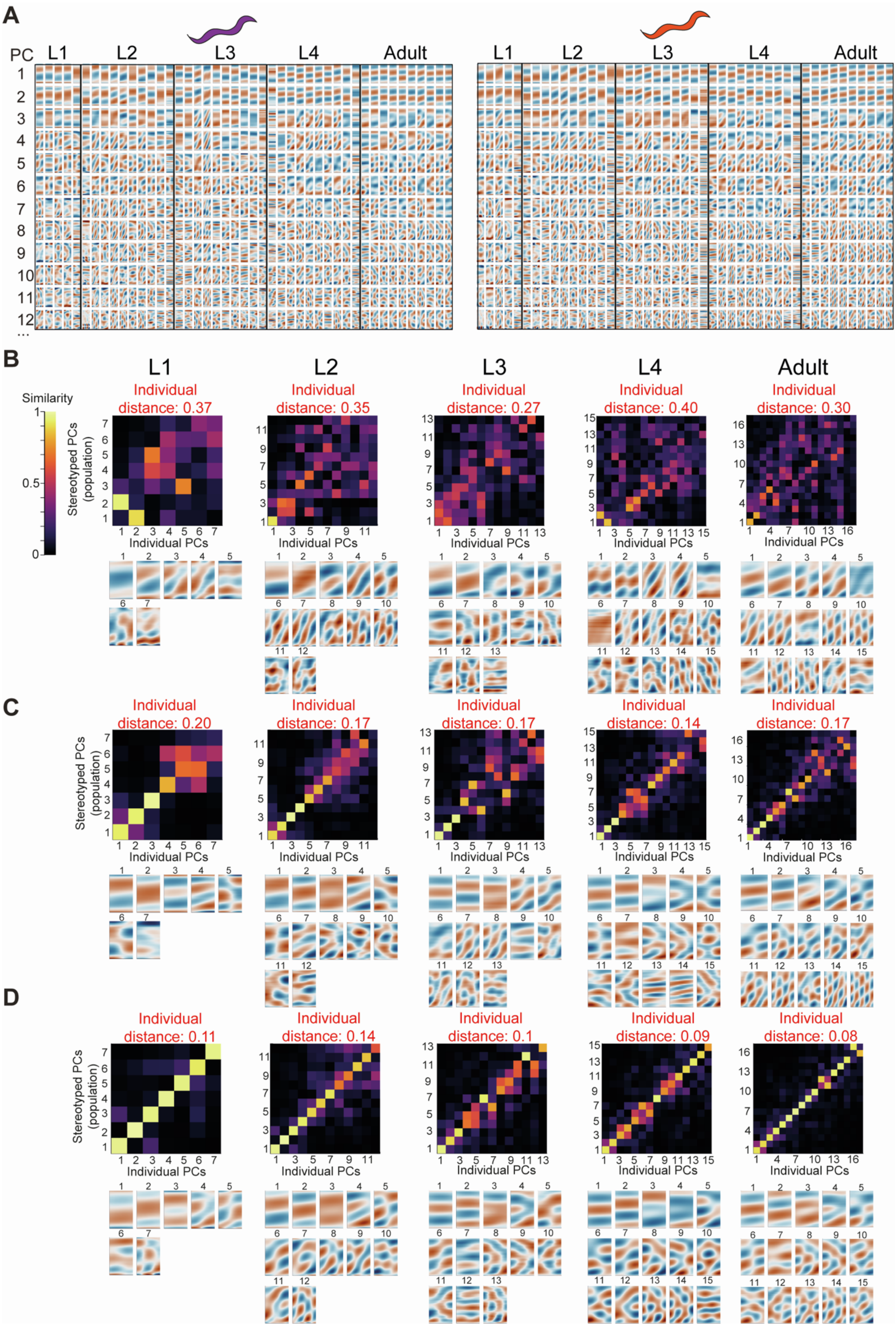

A
PC
L1
L2
L3
L4
Adult
B
Similarity
Stereotyped PCs (population)
Individual PCs
Individual distance: 0.37
Individual distance: 0.35
Individual distance: 0.27
Individual distance: 0.40
Individual distance: 0.30
C
Individual distance: 0.20
Individual distance: 0.17
Individual distance: 0.17
Individual distance: 0.14
Individual distance: 0.17
D
Individual distance: 0.11
Individual distance: 0.14
Individual distance: 0.1
Individual distance: 0.09
Individual distance: 0.08

Figure S3. Related to Figure 3.

Inter-individual variation in behavioral spaces across developmental stages

(A) Examples of developmental trajectories of PCA behavioral spaces from mid L1 stage to adulthood (45 developmental windows), separately generated for single wild-type individuals (see Methods). Shown are the first 12 PCs generated by the PCA. **(B-D)** Examples of similarity matrices between the individual-unique and the population's stereotyped PC modes (top) and the corresponding individual's PC modes (bottom), in animals that showed high (B), intermediate (C) or low (D) behavioral uniqueness during specific developmental time bins. Time windows represented: L1 – 7th time bin, L2 – 7th time bin, L3 – 7th time bin, L4 – 7th time bin, Adult – 5th time bin, out of 10 time bins per developmental stage. Color code in (B-D) marks similarity between different PCs, quantified as the absolute value of the dot product (0-1).

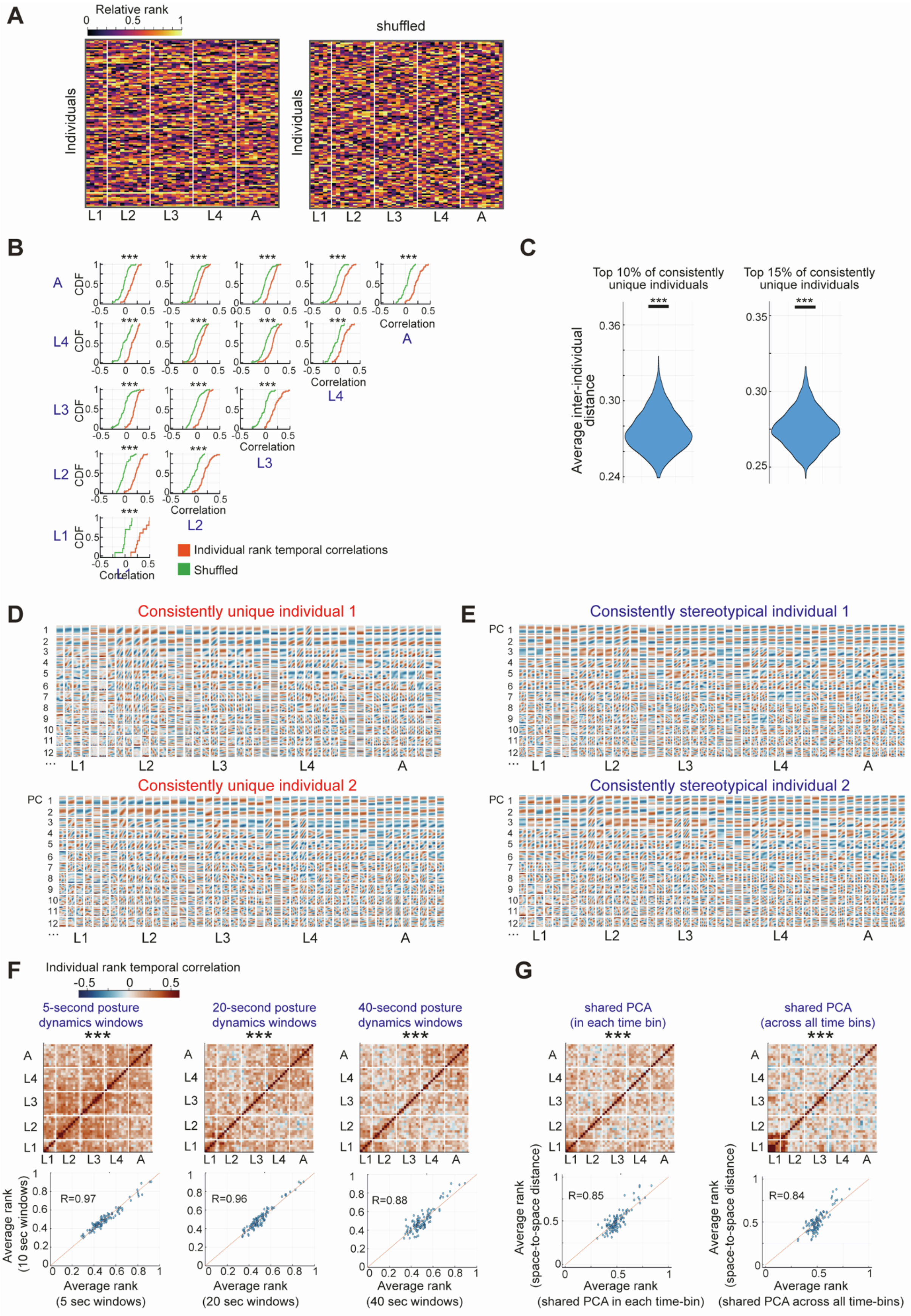
A
Relative rank
shuffled
Individuals
L1 L2 L3 L4 A
B
CDF
Correlation
Individual rank temporal correlations
Shuffled
C
Top 10% of consistently unique individuals
Top 15% of consistently unique individuals
Average inter-individual distance
D
Consistently unique individual 1
Consistently unique individual 2
E
Consistently stereotypical individual 1
Consistently stereotypical individual 2
PC
F
Individual rank temporal correlation
5-second posture dynamics windows
20-second posture dynamics windows
40-second posture dynamics windows
Average rank (10 sec windows)
R=0.97
R=0.96
R=0.88
Average rank (5 sec windows)
Average rank (20 sec windows)
Average rank (40 sec windows)
G
shared PCA (in each time bin)
shared PCA (across all time bins)
Average rank (space-to-space distance)
R=0.85
R=0.84
Average rank (shared PCA in each time-bin)
Average rank (shared PCA across all time-bins)

Figure S4. Related to Figure 4.

Long-term individual consistency in uniqueness levels of behavioral spaces

(A) Left: Relative uniqueness rank of behavioral spaces of wild-type individuals across developmental time windows. Right: A shuffled dataset of individual relative ranks. **(B)** Distributions of temporal correlations between uniqueness rank of wild-type individuals (represented by CDF plots), quantified separately across and within all pairs of developmental stages (orange), compared to a shuffled rank dataset (green). *** P-value<0.001 (comparison to 1000 shuffled datasets) (see Methods). **(C)** Average distance between behavioral spaces among highly unique individuals (top 10% and 15%, based on average rank) across all developmental time windows (black bar) compared to the average distance within random groups of the same size (blue, 1000 runs). *** P-value<0.001. **(D,E)** Individual-specific behavioral spaces across development of consistently unique (D) and stereotypical individuals (E) shown in (Fig. 4E,F). **(F)** Top: Temporal correlations between relative uniqueness rank of behavioral spaces generated from posture dynamics windows of different sizes (5, 20, and 40 seconds), of wild-type individuals with a full trajectory of PCA spaces (see Methods). Bottom: Correlation between average uniqueness ranks of individuals based on PCA spaces generated from posture dynamics windows of different sizes (5, 20, and 40 seconds) and average uniqueness ranks of individuals based on PCA spaces generated from 10 second posture dynamics windows. Each dot represents a single individual. R indicates Pearson correlation coefficient. **(G)** Top: Temporal correlations between relative uniqueness ranks of wild-type individuals within shared behavioral spaces generated from all posture dynamics windows of the population in each developmental time bin (left) or a single shared behavioral space across all time bins (right) (see Methods). Bottom: Correlation between average uniqueness ranks of wild-type individuals within a shared behavioral space generated in each developmental time bin (left) or a single shared behavioral space across all time bins (right) (see Methods) and average uniqueness ranks of individuals based on comparison between the individual-specific and the population's behavioral spaces. Each dot represents a single individual. R represents Pearson correlation coefficient. *** p-value<0.001 in (F,G) for comparison to a shuffled rank dataset.

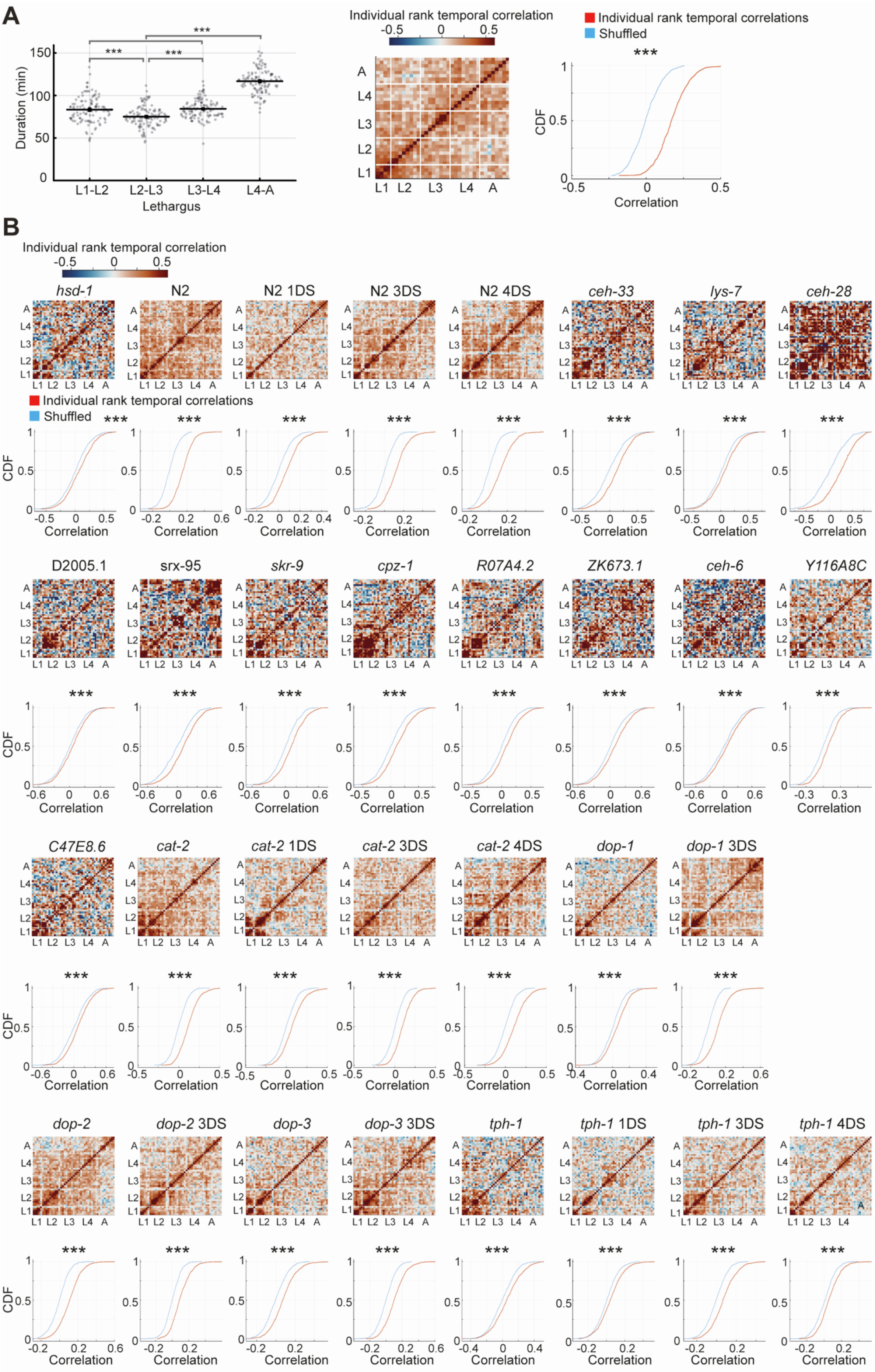
A
Duration (min)
150
100
50
0
***
***
***
L1-L2
L2-L3
L3-L4
L4-A
Lethargus
Individual rank temporal correlation
-0.5
0
0.5
A
L4
L3
L2
L1
Individual rank temporal correlations
Shuffled
CDF
Correlation
B
hsd-1
N2
N2 1DS
N2 3DS
N2 4DS
ceh-33
lys-7
ceh-28
D2005.1
srx-95
skr-9
cpz-1
R07A4.2
ZK673.1
ceh-6
Y116A8C
C47E8.6
cat-2
cat-2 1DS
cat-2 3DS
cat-2 4DS
dop-1
dop-1 3DS
dop-2
dop-2 3DS
dop-3
dop-3 3DS
tph-1
tph-1 1DS
tph-1 3DS
tph-1 4DS

Figure S5. Related to Figure 4 and Figure 6.

Analyses of variation in lethargus periods and temporal patterns of consistency in behavioral uniqueness within multiple populations

(A) Left**:** Durations of lethargus periods across the 4 transitions between developmental stages (see Methods). Each dot represents a single individual. Black lines indicate the mean across the population. *** P-value<0.001 (Mann-Whitney test). Middle: Temporal correlations between relative uniqueness ranks of wild-type individuals following exclusion of the developmental time bins that include the lethargus periods (end and start time bins of each developmental stage). Right: Corresponding CDF plot of distribution of temporal correlations across pairs of analyzed developmental windows (red), relative to CDF plot generated from a shuffled rank dataset (blue). *** P-value<0.001 by bootstrap analysis (see Methods).

(B) Heatmaps represent temporal correlations (Pearson correlation) between relative uniqueness ranks of individuals within the different populations across developmental windows (top), and corresponding CDF plots of distributions of temporal correlations across all pairs of developmental windows (red), relative to CDF plots generated from a shuffled rank dataset of each population (blue) (bottom). *** P-value < 0.001 (FDR corrected) by bootstrap analysis (see Methods). Analyses include individuals with a full trajectory of PCA spaces from mid L1 stage to adulthood (45 developmental windows) (see Methods).

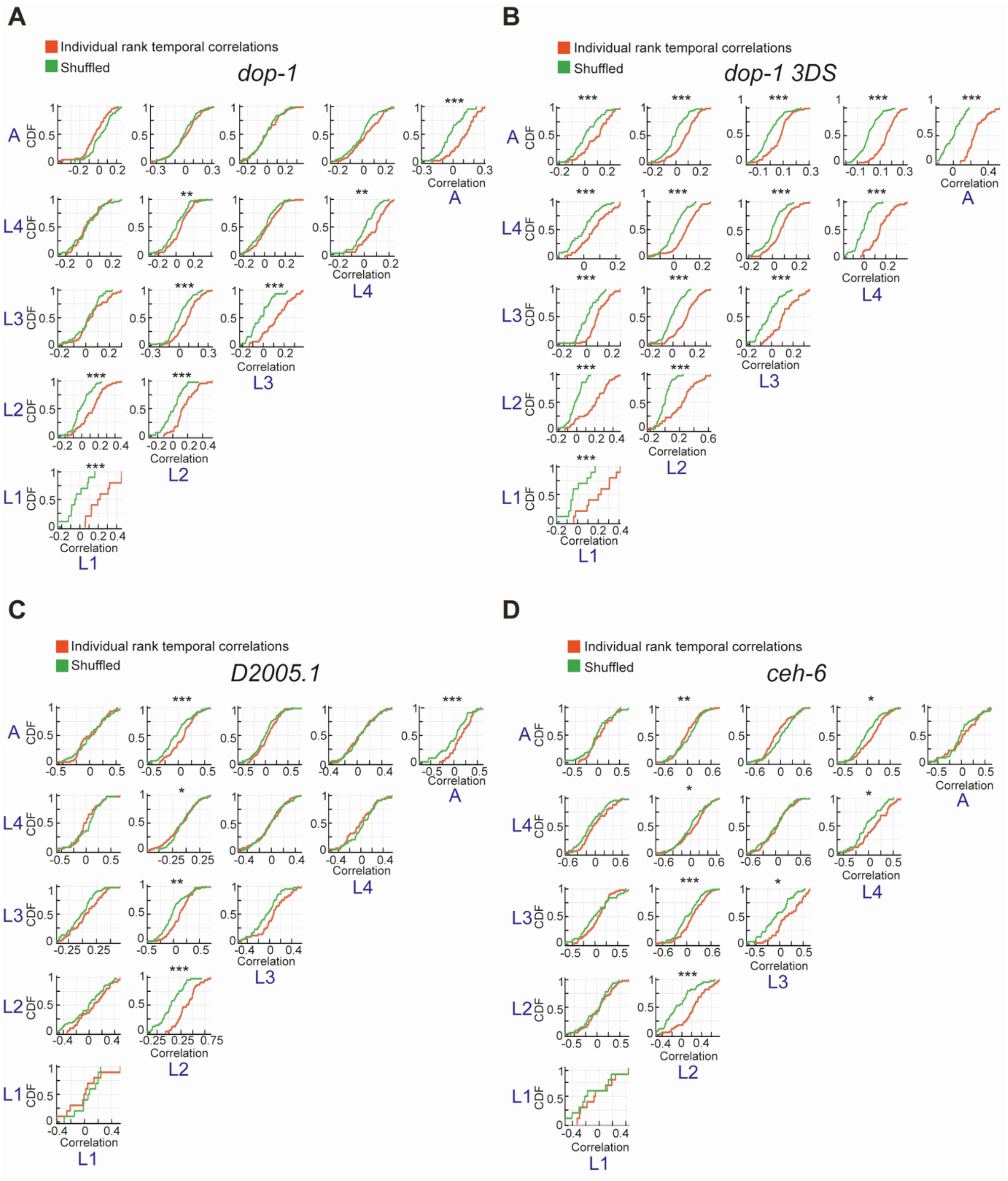
A
Individual rank temporal correlations
Shuffled
dop-1
B
Individual rank temporal correlations
Shuffled
dop-1 3DS
C
Individual rank temporal correlations
Shuffled
D2005.1
D
Individual rank temporal correlations
Shuffled
ceh-6
CDF
Correlation
A
L4
L3
L2
L1

Figure S6. Related to Figure 6.

Non-homogenous temporal patterns of individuals consistency in behavioral uniqueness across development

(A-D) Distributions of temporal correlations (represented by CDF plots) between uniqueness relative rank of *dop-1* (A), *dop-1* exposed to 3 days of early starvation (B), *D2005.1* (C) and *ceh-6* (D) individuals, quantified separately across and within developmental stages (orange), compared to CDF plots generated from a shuffled rank dataset (green). * P-value < 0.05, ** P-value < 0.01, *** P-value < 0.001 (FDR corrected) by bootstrap analysis (see Methods).
